# A precision functional atlas of personalized network topography and probabilities

**DOI:** 10.1038/s41593-024-01596-5

**Published:** 2024-03-26

**Authors:** Robert J. M. Hermosillo, Lucille A. Moore, Eric Feczko, Óscar Miranda-Domínguez, Adam Pines, Ally Dworetsky, Gregory Conan, Michael A. Mooney, Anita Randolph, Alice Graham, Babatunde Adeyemo, Eric Earl, Anders Perrone, Cristian Morales Carrasco, Johnny Uriarte-Lopez, Kathy Snider, Olivia Doyle, Michaela Cordova, Sanju Koirala, Gracie J. Grimsrud, Nora Byington, Steven M. Nelson, Caterina Gratton, Steven Petersen, Sarah W. Feldstein Ewing, Bonnie J. Nagel, Nico U. F. Dosenbach, Theodore D. Satterthwaite, Damien A. Fair

**Affiliations:** 1 Masonic Institute for the Developing Brain, University of Minnesota, Minneapolis, MN USA; 2 Department of Pediatrics, University of Minnesota, Minneapolis, MN USA; 3 Department of Neuroscience, University of Pennsylvania, Philadelphia, PA USA; 4 Penn Lifespan Informatics and Neuroimaging Center, University of Pennsylvania, Philadelphia, PA USA; 5 Department of Radiology, Washington University School of Medicine, St. Louis, MO USA; 6 Department of Psychology, Northwestern University, Evanston, IL USA; 7 Department of Psychology, Florida State University, Tallahassee, FL USA; 8 Department of Psychiatry, Oregon Health & Science University, Portland, OR USA; 9 Department of Medical Informatics and Clinical Epidemiology, Oregon Health and Science University, Portland, OR USA; 10 Knight Cancer Institute, Oregon Health & Science University, Portland, OR USA; 11 Center for Mental Health Innovation, Oregon Health and Science University, Portland, OR USA; 12 Department of Neurology, Washington University School of Medicine, St. Louis, MO USA; 13 Data Science and Sharing Team, National Institute of Mental Health, Bethesda, MD USA; 14 Joint Doctoral Program in Clinical Psychology, San Diego State University, San Diego, CA USA; 15 Joint Doctoral Program in Clinical Psychology, University of California San Diego, San Diego, CA USA; 16 Institute of Child Development, University of Minnesota, Minneapolis, MN USA; 17 Department of Psychological and Brain Sciences, Washington University School of Medicine, St. Louis, MO USA; 18 Department of Neuroscience, Washington University School of Medicine, St. Louis, MO USA; 19 Department of Biomedical Engineering, Washington University School of Medicine, St. Louis, MO USA; 20 Department of Psychology, University of Rhode Island, Kingston, RI USA; 21 Department of Psychiatry, University of Pennsylvania, Philadelphia, PA USA

**Keywords:** Network models, Brain, Databases, Cognitive neuroscience, Neural circuits

## Abstract

Although the general location of functional neural networks is similar across individuals, there is vast person-to-person topographic variability. To capture this, we implemented precision brain mapping functional magnetic resonance imaging methods to establish an open-source, method-flexible set of precision functional network atlases—the Masonic Institute for the Developing Brain (MIDB) Precision Brain Atlas. This atlas is an evolving resource comprising 53,273 individual-specific network maps, from more than 9,900 individuals, across ages and cohorts, including the Adolescent Brain Cognitive Development study, the Developmental Human Connectome Project and others. We also generated probabilistic network maps across multiple ages and integration zones (using a new overlapping mapping technique, Overlapping MultiNetwork Imaging). Using regions of high network invariance improved the reproducibility of executive function statistical maps in brain-wide associations compared to group average-based parcellations. Finally, we provide a potential use case for probabilistic maps for targeted neuromodulation. The atlas is expandable to alternative datasets with an online interface encouraging the scientific community to explore and contribute to understanding the human brain function more precisely.

## Abstract (web summary)

The Masonic Institute for the Developing Brain (MIDB) Precision Brain Atlas is a resource of personalized brain network topographies (*n* = 9,900). It also provides a probabilistic atlas and integration zones across diverse magnetic resonance imaging (MRI) datasets and ages. The atlas increases the reliability of brain-wide association studies (BWAS) and improves targeting for neuromodulation.

## Main

In recent decades, there have been several attempts to generate representations that delineate homogenous functional brain areas into parcellations or networks for use in noninvasive neuroimaging^1,2^. These efforts have led to a series of structure- and function-based parcellations that investigators use for various types of brain-wide association studies (BWAS). These regional parcellations of network descriptions are often based on group-averaged data^1,3–7^. However, considerable interparticipant variability in network topography^8,9^ on the macroscopic scale^10–12^ might reduce BWAS power^13,14^ or the applicability of these parcellations to assist in person-specific interventions^15,16^.

Until recently, limited investigations have attempted to clearly describe individual variation of network-level topographical organization. Although there is some degree of shared patterns of network organization among healthy populations, it is clear that large-scale brain networks show specific deviations from the group organization that are stable^11,17^. Building on prior work using data-driven community detection to identify separable networks in the brain^7,18^, Laumann et al. precisely mapped the network structure in an individual, from whom they had collected more than 14 h of resting-state data^11^^,19^. This approach, termed Precision Functional Mapping (PFM), revealed that although individuals have broadly similar networks to those identified in group averages, specific aspects of the topographical organization of these systems are highly unique.

## PFM provides challenges for traditional data acquisitions

Precisely mapping an individual’s brain may require upwards of 40–60 min of resting-state data^9,11^. However, the collection of 40–60 min worth of data per participant is a burden to the participant and expensive for the investigative team and therefore creates limitations for widespread adoption. Extended collection of resting data creates additional obstacles for studies in childhood development and disease research, where a resting-state session is typically limited to shorter durations. For example, the Adolescent Brain Cognitive Development (ABCD) study (11,987 participants enrolled at baseline) was designed to determine biological and environmental factors that impact brain function by collecting resting-state and task functional magnetic resonance imaging (fMRI) data in participants representative of the United States population at of 9–10 years old and biennially for 10 years^20,21^. Although ABCD will provide an impressive resource for describing individual variation in network organization over time, ‘only’ 20 min of resting-state data are collected per participant, which may reduce the ability to maximize the precision of the individualized connectome across all participants. However, the shorter resting-state dataset is still valuable for precision mapping using new ‘supervised’ methods^22,23^ that create individual-specific networks that may only be marginally less precise. Furthermore, as task activity only adds a relatively small amount of variance to global resting-state brain organization^24^, the additional task fMRI data (40 min) per participant can be used to generate individual-specific networks using similar amounts of data as prior reports^9,11^. The combination of a relatively large sample from ABCD and relatively long blood-oxygen-level-dependent (BOLD) data collected from each participant provides the unique opportunity to provide individual network topographies and to produce a probabilistic atlas of functional networks.

## Probabilistic atlases are mostly relegated to structure

Historically, probabilistic atlases in neuroimaging have been structural, not functional. For example, the standard Montreal Neurological Institute (MNI) and other widely used brain atlases^25–27^ are derived from hundreds of magnetic resonance imaging (MRI) scans for image registration. These procedures often use probabilistic weights to attempt to delineate anatomical structures, such as the cortex^25^, amygdala^28^, basal ganglia^29^ and brainstem nuclei^30^. Probabilistic volumes for subcortical structures are also often associated with these atlases, providing probabilistic-based regions of interest (ROIs). Just as these methods have vastly improved standard structural registration and segmentation, functional probabilistic maps may also be leveraged to create individual-specific functional mappings in group-level studies that would normally lack sufficient amounts of data for individual-specific mapping. Moreover, these maps could enhance neuronavigation for targeted brain stimulation based on functional information rather than solely relying on anatomical landmarks, benefiting situations without resting-state data or access to community-detection techniques for precise brain mapping.

## The MIDB Precision Brain Atlas provides personalized maps and derivatives

Building on recent reports using probabilistic mapping approaches to resting-state functional connectivity MRI^22^, we implement the following various methods of network identification: Infomap (IM)^9,31^, template matching (TM)^22,23^, non-negative matrix factorization (NMF)^8,32^ and an original overlapping network method, Overlapping MultiNetwork Imaging (OMNI) mapping, to generate individual-specific network mappings, along with a population network probability atlas from resting-state fMRI (rs-fMRI) data from the ABCD study. The original overlapping network technique supports evidence that certain networks, in particular the default mode^33^, may have regions with subsystems^34,35^. The highly reproducible probabilistic atlases enable the derivation of ROI sets that reflect the variation of brain topography of individuals.

Our resource introduces the MIDB Precision Brain Atlas featuring individual-specific networks, population probabilistic maps, individual integrative zones and population integrative zones. We encourage contributions of personalized networks and probabilistic maps to the MIDB Precision Brain Atlas. Alongside ABCD probabilistic maps, we are sharing additional maps across additional ages from the HCP-D (The Lifespan Human Connectome Project Development) Project^36^ and maps generated in Dworetsky et al.^22^ from a Washington University dataset^37^, a Dartmouth dataset^5^, the Midnight Scan Club (MSC) dataset^9^ and the Human Connectome Project (HCP) dataset^38^. Notably, other groups have developed analogous network mapping techniques^2,6,39,40^, which require task-based, or multi-session resting-state data, which we hope to add to this resource in the future. The atlas includes a user-friendly downloader tool with adjustable thresholds for network assignments and functional integration zones (https://midbatlas.io/). As a resource for the scientific community, it will enable systematic exploration of network topography contributions to human cognition and behavior.

## Results

While the MIDB Precision Brain Atlas currently contains multiple child and adult datasets (https://midbatlas.io/), as noted, here we focus on the ABCD cohort (Extended Data Table 1). This specific example demonstrates the rationale for individual-specific topography, quantifies within-participant reliability and showcases the atlas’s utility.

### ABCD cohort demographics

The ABCD data were divided into two large cohorts (discovery Cohort ABCD-1 (*n* = 5,786) and replication Cohort ABCD-2 (*n* = 5,786)) and one smaller test cohort (Cohort ABCD-3 (*n* = 300)), matched on multiple demographics (Extended Data Table 2; that is, the ABCD reproducible matched samples (ARMS) from the ABCD BIDS Community Collection (ABCC)^13^). From these initial groups, participants with at least 10 min of low-motion data were chosen to test replication (group 1, *n* = 2,995 and group 2, *n* = 3111) based on a framewise displacement (FD) <0.2 mm, which retained similar proportional demographics to that of the full cohort (Extended Data Table 3 and Supplementary Fig. 1). Group 3 (*n* = 164 with available processed MRI data) was used to build the network templates for the TM procedure described below. Groups 1 and 2 were test groups used to validate the community detection methods.

### Individual-specific mapping is robust across techniques

We first sought to establish that each method produces consistent within-participant networks by demonstrating that a given participant is distinguishable from the group. Individual-specific networks were successfully mapped using the following methods: IM^9,41^, TM^22,23^ and NMF^8,32^. For all participants in groups 1 and 2, we created individual-specific network maps by generating dense connectivity matrices (91,282 × 91,282 grayordinates) from exactly 10 min of resting-state data randomly sampled from the full length of data below a FD threshold of 0.2 mm (Supplementary Fig. 3a; Methods). This allowed for direct participant-to-participant comparison despite differences in movement characteristics within the MRI scanner between participants. Identical matrices were used for each individual as inputs for both the IM and TM procedures (see below).

The IM algorithm uses information theory to map networks by modeling the flow between nodes. It implements a random walk strategy using the connection weights to minimize the bits (using Huffman coding) necessary to identify each node’s (that is, grayordinate’s) network. Each connectivity matrix was thresholded to discrete percentiles of connections (or edges), and then IM identified community structure at each threshold (Methods). Finally, we generated a consensus across the edge densities to (1) ensure that similar communities are identified among the groups; and (2) accurately assign distinct communities to larger networks and (3) provide brain coverage, as in previous work^9^.

The TM algorithm assigns each grayordinate to a network by comparing the whole-brain connectivity of the grayordinate to a series of network templates observed in the group^5,22^, a method developed by Gordon et al.^23^. Supplementary Fig. 3 shows the technique used to establish individual-specific networks using TM. Using group 3 participants, templates were generated for each network. This was done by using network templates previously identified with IM with an average dense connectivity matrix from 120 adult participants^9,11^. We then conducted a seed-based correlation whereby the motion-censored (see below) resting-state data for each grayordinate are correlated with the average resting-state signal for the respective network. To perform TM, we used the network templates generated with group 3 to measure the extent to which the whole-brain connectivity of each grayordinate resembles the connectivity pattern of the template network for each participant in groups 1 and 2 (Supplementary Fig. 3a; using *η*^2^). Supplementary Fig. 4 shows the network templates that were used, which correspond closely with networks that have been previously identified within the literature^32,42^. All of these individual-specific maps are available on the National Institute of Mental Health (NIMH) Data Archive (NDA) or ABCD’s future release platform via the ABCC^13,43^. Future distribution locations will be available and updated via the ABCC ‘Read the Docs’ (https://collection3165.readthedocs.io/).

### Network mapping methods show high intraparticipant reliability

**Fig. 1:**
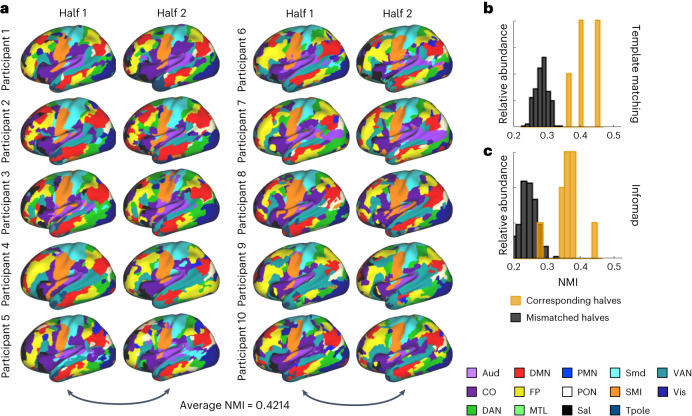
Example of precision maps of ABCD participants using TM. **a**, Example of networks determined by the TM procedure for participants with at least 20 min of low-motion resting-state data. Resting-state time series were split in half, and networks were obtained for each half (*n* = 10). Only the left hemisphere is shown for visualization purposes, but networks were also identified in the right hemisphere, subcortex and cerebellum. **b**, The NMI was calculated between participants’ own halves (gold bars) and others in the split-half group (gray bars) using TM. **c**, We also generated network maps using the IM procedure and performed an identical NMI comparison.

To establish that these methods can reliably generate individual-specific network maps using limited amounts of data (that is, 10 min of motion-free data), we used split-half reliability analysis to demonstrate that similar network maps are generated when using the first versus second half of a participant’s time series (Fig. 1). We conducted split-half reliability analyses for each method in ten participants from group 1 who had the longest duration of low-motion quality data, exceeding 20 min. To assess network reliability, we used normalized mutual information (NMI). We compared the topographic similarity of network maps generated within-participant (intraparticipant; first half of data versus second half of data) to network maps generated between different participants (interparticipant). The distribution of the NMI between intraparticipant halves was compared against a null distribution of the NMI between interparticipant halves (Fig. 1b,c). For both TM and IM, intraparticipant NMI was significantly higher than interparticipant NMI (TM: *t*(9.31) = 11.87, *P* = 3.1079 × 10^−7^; IM: *t*(9.607) = 9.049, *P* = 2.6109 × 10^−6^; unequal variance assumed, one-tailed). Comparing methods, TM displayed significantly higher similarity both between halves of data from the same participant (mean NMI for TM = 0.421; IM = 0.370, *t*(18) = 2.951, *P* = 0.009) and different participants (*t*(358) = 16.0315, *P* = 5.60 × 10^−^^44^; equal variance assumed, two-tailed; Fig. 1b versus Fig. 1c (gold bars)). TM had a higher between-group similarity compared to IM when we used group-averaged connectivity matrices (Supplementary Fig. 6). Overall, despite intraparticipant variability, these data highlight that networks generated by both methods are highly specific to each individual.

### Personalized maps are reliable with less time using TM

We assessed the minimum low-motion resting-state data required to produce individual-specific network maps using MSC data^9^. Similar to ABCD, we performed split-half reliability analysis for networks generated by the TM procedure using interleaved concatenated sessions (‘Data requirements for network specificity’). Individual-specific networks were generated from 1 to 20 randomly sampled noncontiguous minutes of low-motion data (ten times each) from one-half of each participant’s data and compared to networks generated from the second half of data using NMI. We demonstrate that individual-specific maps produced by TM, even with relatively few minutes of low-motion data, reliably resemble the individual-specific network maps observed with 10 min of low-motion data (Supplementary Fig. 5). It should be noted that randomly sampled data from longer-duration acquisitions likely improve reliability (increase in correlation up to 0.04) by reducing time-series autocorrelation^11^. While this split-half analysis was in adults, a parallel analysis in adolescents showed similar reliability, aligning with our prior NMF work (Supplementary Fig. 19)^8,44^.

### Probabilistic maps are reliable within and across techniques

**Fig. 2:**
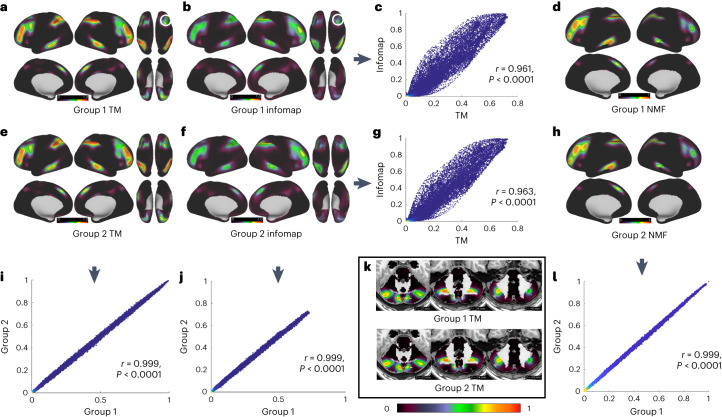
Example of network probability. **a**,**b**,**d**–**f**,**h**, An example of network probability for the frontoparietal network using TM (**a**,**e**), IM (**b**,**f**; surface only) and NMF (**d**,**h**; surface only) procedures with single network assignment. **i**,**j**,**l**, The between-group correlation for TM (**i**), IM (**j**) and NMF (**l**), respectively. **c**,**g**, The correlation between methods for ABCD-group 1 (**c**) and ABCD-group 2 (**g**), respectively. For additional probability maps, see Supplementary Fig. 8. **k**, Network probabilistic map for the frontoparietal network within the cerebellum. White circles in **a** and **b** highlight similar probabilistic functional asymmetries in the SMA across methods. Each dot in **c**, **g**, **i**, **j** and **l** represents one grayordinate. Independent Pearson’s correlations were conducted between groups 1 and 2 for each network. The color in the scatterplot is the probability density estimate based on a normal kernel function. SMA, supplementary motor area.

Next, we illustrated the extent to which each grayordinate participates in each network across both ABCD-group 1 and ABCD-group 2. Using the individual-specific mapping methods described above, we generated probabilistic maps in both ABCD-group 1 and ABCD-group 2 to highlight replicable network probabilities between the groups. Individual-specific maps were generated for each participant within ABCD-group 1 and ABCD-group 2 (Supplementary Figs. 8 and 9), and then the probability of network observation was calculated for each grayordinate for each group separately (Fig. 2a,d). To test replication between groups (Fig. 2i,j) and methods (Fig. 2c,d), we correlated nonzero values of probabilistic maps. For example, the frontoparietal network (Fig. 2a,e) shows remarkable replicability (*r* = 0.9996; Fig. 2i) between groups, even with respect to functional asymmetries. Note how the frontoparietal representation in the dorsolateral prefrontal cortex (DLPFC) in the right hemisphere compared to the left is clearly present in both groups. These maps highlighted discrete cerebellar nuclei communicating extensively with the frontoparietal network (Fig. 2k), aligning spatially with previous observations^45^.

To confirm that the probabilistic network representations observed in Fig. 2a,e are not simply the product of the TM method, we used the same data to generate probabilistic maps using a robust community detection method for large-scale neuroimaging data, IM (Fig. 2b,f)^9,11^ and NMF (Fig. 2d,h)^8,46^. We compared methods by correlating the probabilistic maps between IM and TM for ABCD-group 1 (Fig. 2c) and ABCD-group 2 (Fig. 2g), respectively. Cross-method correlation analysis between NMF and other methods was not performed due to the differing number of unique networks. Nevertheless, NMF probabilistic maps demonstrate very high correlation between groups (Fig. 2l; *r* = 0.9996, *P* < 0.0001; Supplementary Table 3).

Probabilistic network topography remains highly conserved across methods (albeit overall probability is slightly reduced in IM), suggesting that the supervised method produces nearly identical networks to an unsupervised approach (frontoparietal network (nonzero correlation)—group 1 (TM to IM): *r*(91282) = 0.951, *P* < 0.0001; group 2 (TM to IM): *r*(91282) = 0.954, *P* < 0.0001) even retaining the aforementioned asymmetries. Supplementary Table 1 provides the correlation between methods for the remaining networks (median between-method correlation: ABCD-group 1 = 0.937 and ABCD-group 2 = 0.936). Visual comparisons for all networks are provided (Supplementary Fig. 8). In addition, we generated probabilistic maps for each network, using 10 min of low-motion data from the cerebral cortex only (that is, excluding subcortical nuclei and cerebellum; Supplementary Fig. 9).

To demonstrate the generalizability of probabilistic maps to a broader age range, we conducted an identical split-half comparison using a cohort of children 8–13 years old (half 1, *n* = 81 and half 2, *n* = 81) from the HCP dataset (Supplementary Fig. 17). Again, we demonstrate that the correlation between probabilistic maps between each group is high (average correlation = 0.9780 ± 0.008; Supplementary Table 6), and the similarity to the ABCD probabilistic maps was high (Supplementary Table 7).

To assess the impact of including task data to generate probabilistic maps, the same between-method comparison was performed using concatenated rest and task data instead of rest alone (Supplementary Fig. 10). Including task data provided up to an additional 40 min of data per participant. The decision to include task data in addition to rest was motivated by recent research that suggests that the proportion of variation in edges explained by individual features of connectivity was substantially higher than cognitive variation induced by tasks. In addition, measures of topography as opposed to topology are also less influenced by task-related activity^24^. We observed strong replication between groups (median between-method correlation: ABCD-group 1 = 0.900 and ABCD-group 2 = 0.900), but crucially, the probability maps are nearly identical to networks generated from resting-state data alone, despite differing amounts of data used to generate the maps (Supplementary Figs. 8 versus 10 and Supplementary Table 4). Supplementary Fig. 10 shows the probability map for the network using TM with a single network assignment, similar to what was shown in Supplementary Fig. 9 except with task data included (Methods). This suggests that the contribution of task-induced, activation-related, hemodynamic responses does not appreciably affect global network topographic organization, as noted above and which others have previously proposed^24,47^.

### Probabilistic based ROIs improve reliability in BWAS

**Fig. 3:**
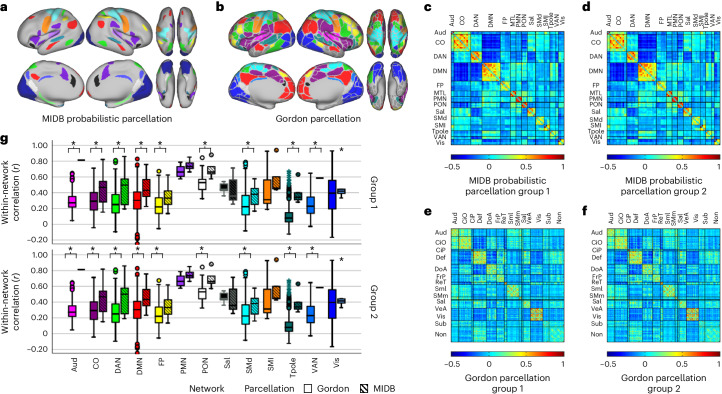
Comparing connectivity using probabilistic ROIs and Gordon ROIs. **a**, MIDB probabilistic parcellation (75% probability of network consensus using the TM). **b**, Gordon parcellation. Parcels are colored according to network assignment. Similar colors were used between parcellations where possible. **c**–**f**, Connectivity matrices were generated using the MIDB probabilistic parcellation (**c**,**d**) and the Gordon parcellation (**e**,**f**) for ABCD-group 1 (**c**,**e**) (*n* = 2,995) and ABCD-group 2 (**d**,**f**) (*n* = 3,111). **g**, In total, 9 of 13 shared networks showed significantly higher within-network connectivity in the MIDB probabilistic parcellation compared to the Gordon parcellation. Open boxes represent Gordon parcellation; striped boxes represent MIDB probabilistic parcellation. *T* tests were conducted between methods using within-network connections using group average connectivity matrices (Aud, d.f. = 275; CO, d.f. = 844; DAN, d.f. = 522; DMN, d.f. = 896; FP, d.f*.* = 276; PMN, d.f. = 14; PON, d.f. = 32; Sal, d.f. = 19; SMd, d.f. = 716; Sml, d.f. = 32; Tpole/unlabeled, d.f. = 1079; VAN, d.f. = 252; Vis, d.f. = 745; the number of ROIs and therefore the number of degrees of freedom are identical for groups 1 and 2). *P < 0.05 (Benjamini–Hochberg corrected, two-tailed). Boxplots show median and IQR (box size). The maximum and minimum whiskers represent *Q*3 + 1.5 × IQR and *Q*1 − 1.5 × IQR. IQR, interquartile range; CiO, cingulo opercular; CiP, cingulo parietal; Def, default; DoA, dorsal attention; FrP, frontoparietal; ReT, retrosplenial temporal; Sml, somatomotor lateral; SMm, somatomotor medial; VeA, ventral attention; Sub; subcortical; Non, no assignment.

Recent evidence suggests that connectivity-based BWAS show limited predictive power when using whole-brain associations with small samples^14^; therefore, we wanted to test if omitting network topographies that are highly variable would improve group reliability when we only used commonly observed network locations. Using the resting-state probabilistic maps, we generated a set of network labels to examine connectivity among brain regions that are highly homogenous across participants (Supplementary Fig. 3d). Figure 3 shows the regional network composition and connectivity matrix across both ABCD groups at a 75% threshold (that is, a consensus network map for which at least 75% of the participants were assigned to a respective network). Using these regions of high consensus (80 parcels; Fig. 3a), we produced a parcellated connectivity matrix for each participant. The strength of the within- and between-network connectivity for each cohort was calculated using the MIDB probabilistic parcellation (Fig. 3c,d) versus the Gordon parcellation (Fig. 3e,f), one of the most widely used parcellation schemas in adults, children and adolescents^14,48^, which are based on a population average^5,49^. We observed a significant correlation between the average functional connectivity for each group (Fig. 3c–f; Pearson’s *r* (upper triangle)—TM: *r*(3.16 × 10^3^) = 0.998, *P* = 0; Gordon parcellation: *r*(6.17 × 10^4^) = 0.996, *P* = 0). Compared to Gordon parcellation based on group averages, the MIDB probabilistic parcellation set provides increased within-network connectivity strength between the two group matrices (Fig. 3g; average within-network connectivity—group 1, Gordon: 0.3421 ± 0.1467, TM: 0.5208 ± 0.149, *t*(24) = −3.0801, *P* = 0.0026; group 2, Gordon: 0.3421 ± 0.1467, TM: 0.5189 ± 0.149, *t*(24) = −3.0402, *P* = 0.0028). This increase in connectivity strength is likely due to only including regions with consistent network assignment across the population, and therefore have inhomogeneous connectivity.

**Fig. 4:**
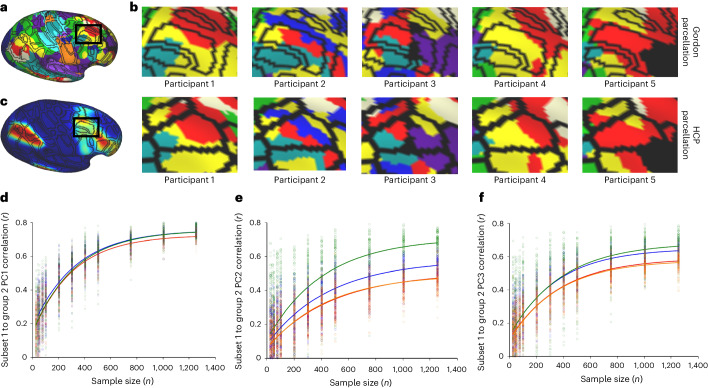
Neural networks have unique topographies that confound conventional ROI sets. The black lines indicate the boundaries of the parcels. **a**, The dorsolateral aspect of the frontal lobe demonstrates that parcels may belong to one of several potential networks. **b**, An example of ten individuals’ neural networks with the Gordon parcellation (top row) and HCP parcellation (bottom row) overlaid. Frontoparietal is shown as yellow. **c**, The frontoparietal probabilistic map indicates inhomogeneity in network topography among the population. **d**–**f**, Subset reliability analysis showing that using the MIDB probabilistic parcellation improves signal-to-noise in group-level predictions relative to the Gordon parcellation. Blue circles/lines indicate intergroup correlation for each random subset using the MIDB probabilistic parcellation. Red circles/lines indicate intergroup correlation for each random subset using the Gordon parcellation. Green circles/lines indicate intergroup correlation for each random subset using the integration zone parcellation. Orange circles/lines indicate intergroup correlation for each random subset using 80 randomly selected parcels from Gordon parcellation. Data were fitted with an exponential rise-to-maximum equation. Please note red and orange fitted curves are nearly identical, which obscures visual discernment.

We examined the added reliability of probabilistic ROI sets in brain–behavior correlations. Conventional ROIs sets that apply the same network assignment to the parcellation schema to all individuals have the potential to dilute the effects that specific brain regions have on behavior. For a given region of interest, several networks may include a given region, and furthermore, the same location may belong to different networks in any given individual (Fig. 4a–c). Using Bayesian probabilistic principal components (PC) analysis^50^ to extract three cognitive traits from ARMS-1 and ARMS-2 reflecting general cognitive ability, executive function and working memory^13,51^, we examined subset reliability using either the Gordon or MIDB probabilistic parcellation, sampling a random subset of group 1 participants. We correlated each element of the connectivity matrix from each subsample with each behavioral factor. We then correlated the brain–behavior correlations from each subsample in group 1 with the brain–behavior correlation using all group 2 participants to serve as the ‘ground truth’ (‘Brain–behavior associations using subset reliability’). The MIDB probabilistic parcellation provided only a modest increase in reliability in general cognitive ability compared to using the Gordon ROIs at all sample sizes. However, for the components of learning/memory and executive function, we observed an increase in reliability (Fig. 4d–f; Cohen’s *d* with 1,250 participants: PC1 = 0.909, PC2 = 1.605 and PC3 = 1.865). A subset of 873 (PC1), 702 (PC2) and 675 (PC3) participants using the MIDB probabilistic parcellation showed the same intergroup brain–behavior correlation observed with 1,250 participants using Gordon parcellation (Fig. 4d–f). Furthermore, to control for the difference in the number of ROIs, we performed an intergroup correlation for each random subset using 80 randomly selected parcels from Gordon parcellation (orange circles), which showed even more robust findings. While these results do not reduce the necessity for large sample sizes in BWAS^52,53^, this offers increased reliability for targeted questions.

### Revealing an overlapping network structure

**Fig. 5:**
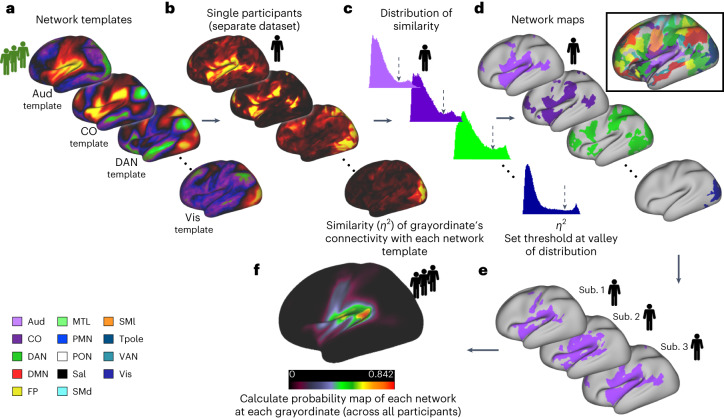
Method for detecting overlapping networks using OMNI mapping. **a**, A series of network templates were generated using an independent group of participants (ABCD-group 3). **b**, For each participant, the similarity at each grayordinate (using *η*^2^) was calculated to each of the network templates shown in **a**. **c**, We set a threshold (dashed arrow) for each network, based on the observed local minimum between peaks of bimodal distribution of *η*^2^. **d**, Grayordinates that had *η*^2^ values that were above the threshold were then assigned that network label. All overlapping networks for an example participant are shown in the inset. **e**,**f**, After this procedure is performed for all participants, we calculate a probabilistic map for each network (only the Aud is shown).

**Fig. 6:**
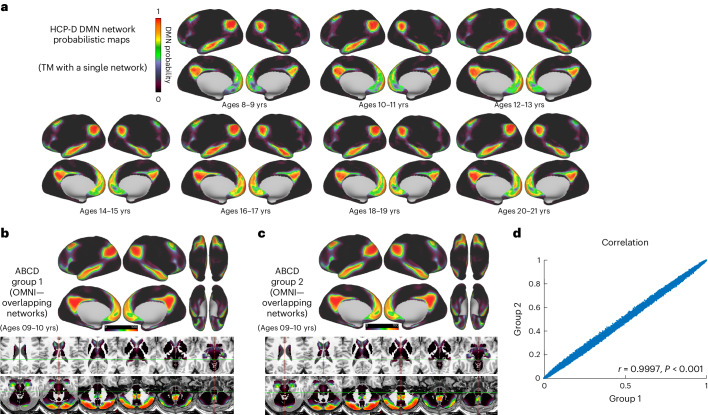
Probabilistic map consistency across ages. **a**, Probabilistic maps from adolescence to adulthood. Here we show the DMN probabilistic map. Please note additional probabilistic maps are available for additional age groups from the HCP-D study (https://midbatlas.io/). **b**–**d**, Network probability maps using OMNI mapping. At each grayordinate, the probability of observing each network was calculated for ABCD-group 1 and ABCD-group 2. Here the DMN is shown as an example. **d**, The correlation of the probability maps depicted in **b** and **c** (excluding zeros; mismatched zeros = 0.033%). See Supplementary Fig. 9 for additional networks.

Many connectivity studies assume that a given grayordinate (or voxel) participates in a single network. However, evidence suggests that some brain regions participate in multiple networks^54^ or demonstrate nested or hierarchical structures that can be better described when allowing communities to overlap^55^. For example, neurons that respond to multimodal stimuli likely participate in multiple networks^56,57^. The default mode in particular retains distinct subsystems that occupy shared cortical regions^34,35^. Our OMNI mapping extends the TM procedure, allowing networks to overlap (Fig. 5)^58,59^. Instead of using a ‘winner-take-all’ labeling, we quantified the similarity of each grayordinate’s BOLD signal to each template network by setting a data-driven threshold based on the observed local minima in the bimodal distribution of *η*^2^ values for each network (Fig. 5c,d). This technique reveals secondary and tertiary (and so forth) networks that communicate with a particular grayordinate that would otherwise be missed by only identifying the primary network. We further quantified the specificity of the overlapping networks from OMNI mapping by comparing the resultant networks from the ten participants in the ABCD test cohort to each other by calculating the NMI between each split half (Supplementary Fig. 7). For each network, we observed similar regions of high probabilistic similarity between ABCD-group 1 and ABCD-group 2 for each of the networks measured (Fig. 6b–d and Supplementary Fig. 11) using OMNI mapping.

### Integration zones are revealed by overlapping networks

After generating the overlapping networks for an individual, we averaged the number of networks observed at each grayordinate across the group to examine the extent to which networks overlap in the population. Regions that demonstrate a high degree of overlap are thought to facilitate communication between networks^59,60^.

**Fig. 7:**
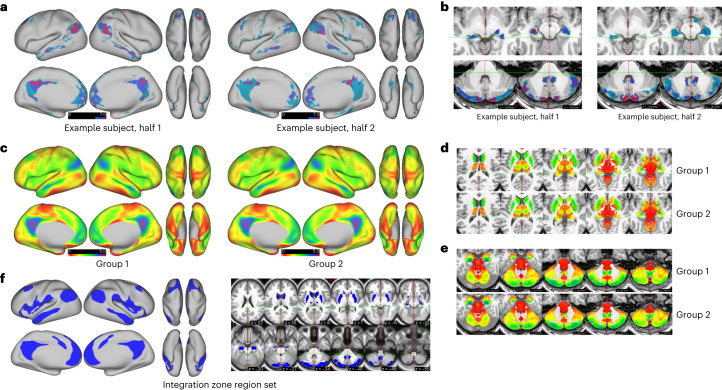
Identifying regions with multiple overlapping networks using OMNI mapping. **a**,**b**, An example of regions identified on the cortex, subcortical nuclei (**a**) and cerebellum that have five or more networks overlapping in an individual participant (image has been thresholded for visualization purposes) (**b**). **c**–**e**, The number of networks that overlap at each grayordinate for ABCD-group 1 and ABCD-group 2. The hippocampi and the posterior cerebellum (in particular the spinocerebellum) also demonstrate high network overlap. **f**, Brain-wide maps of the average number of overlapping networks for ABCD-group 1 (shown in **c**) were thresholded at 2.2 networks to generate an integration zone region set.

Split-half reliability was calculated in the same manner with the ten ABCD participants mentioned previously. Overlapping regions showed high reliability within individuals (average real NMI = 0.4847 ± 0.411 s.d.; null NMI = 0.3287 ± 0.327 s.d., *t*(9.644) = 11.783, unequal variance, *P* = 4.84 × 10^−7^), which was overall greater than using a single network assignment. In addition, we quantified the number of networks detected at each grayordinate. Figure 7a demonstrates that, within a given participant, some integrative zones can even show 8–10 networks converge in regions such as the posterior parietal cortex, precuneus and posterior cerebellum, revealing a complex structure of internetwork communication.

Furthermore, integration zones across the population are highly reliable. The number of networks detected at each grayordinates was calculated for ABCD-group 1 and ABCD-group 2 (Fig. 7c). We found that the integration zones across the population were highly reliable (*r*(91282) = 0.9994, *P* < 0.001). We observed that regions with the highest number of networks closely resembled the default mode network (DMN; Fig. 7), including regions such as the parieto-occipital junction, middle temporal gyrus, posterior cingulate cortex (PCC)/precuneus, hippocampus and the posterior aspect of the posterior cerebellum, consistent with prior work in adults^47^.

Integration zones yielded more reproducible executive function brain-wide associations compared to both the MIDB probabilistic and Gordon parcellations in our subset reliability analysis (Fig. 4d). To ensure that the improvement in reproducibility was not due to fewer ROIs in the integration zone parcellation, we performed a subset analysis on the Gordon parcellation, randomly sampling the same number of ROIs as integration zones. We found that the rise-to-maximum of the randomly sampled Gordon parcellation was nearly identical to the complete Gordon parcellation (Fig. 4 and Supplementary Table 6).

### Probabilistic maps for brain stimulation as a use case

**Fig. 8:**
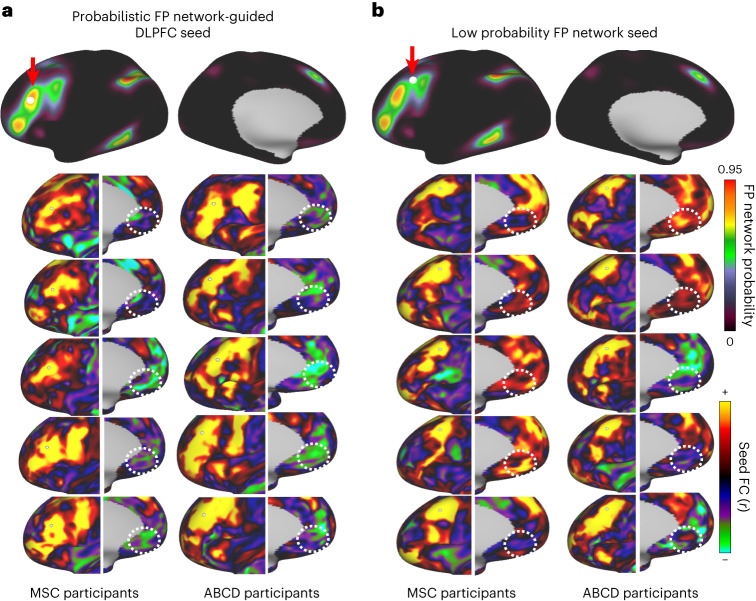
Probabilistic map-guided seed-based correlation. A seed-based correlation was conducted with five MSC and five ABCD participants. **a**, A seed was placed at the DLPFC (as defined by the MIDB frontoparietal probabilistic map) and connectivity to the subgenual cortex was examined (dotted white circle). Note that, in most participants, the connectivity to the frontoparietal network was anticorrelated with the subgenual cortex (green–blue). **b**, When the seed was placed in a region with a low probability of belonging to the frontoparietal network, connectivity to the subgenual cortex was inconsistent. White circles indicate the location of the subgenual gyrus (subgenal MNI coordinates = ±5, 25, −10 (refs. ^97,98^)).

For investigators where a consistent measure of seed-based functional connectivity is used to guide neuromodulatory therapies, such as transcranial magnetic stimulation (TMS), probabilistic functional maps provide a measurable means for targeting a network. Similar to findings discussed in refs. ^16,61,62^, we demonstrate that a seed placed within a region of high network probability (0.75 probability of frontoparietal) within the DLPFC showed consistent anticorrelation with the subgenual cortex, both in the MSC and ABCD participants (Fig. 8a). However, when the seed was moved slightly outside of the region of high network consensus to a region with high network heterogeneity (0.35 probability), the correlation with the subgenual cortex was inconsistent (Fig. 8b). This suggests that the MIDB probabilistic parcellation allows investigators to quantify the confidence of the spatial location of a network of interest and inform targets that could be used in future therapeutic brain stimulation, in situations where personalized network maps are not available.

### The MIDB Precision Brain Atlas

The MIDB Precision Brain Atlas includes an online tool (https://midbatlas.io) with publicly available personalized MRI maps, probabilistic maps and integration zones for various methods and datasets described here and elsewhere^13^. Data are provided in surface and volume where available. Finally, the resource allows for the inclusion of community-generated datasets as well.

## Discussion

Investigations into brain function, especially in developmental studies, require confidence in structure- and function-based parcellations that consider the vast heterogeneity in functional topography from person to person. The MIDB Precision Brain Atlas provides an invaluable resource to explore the brain function for basic and clinical research that accounts for this individual variation in network topography.

The inaugural MIDB Precision Brain Atlas draws from various public datasets and includes over 9,950 participants from the ABCD and HCP studies, along with other prominent datasets. It also features associated probabilistic and integration zone maps, replicated for about 9,000 participants using concatenated tasks and rest data. The MIDB Precision Brain Atlas online repository allows users to customize ROI sets by adjusting the probability threshold. We encourage exploring this collection of individual precision maps, probabilistic maps and integrative zones to understand how individual topographic variation influences traditional network mapping and population-wide network topology in complex behaviors.

### Improving reliability in neuroimaging

Noise in BWAS includes sampling variability and random BOLD fluctuations^13,14^. Large-scale datasets, as shown here, can offer a reasonable personalized topography approximation but might show more variability than densely sampled cohorts like MSC. Ignoring individual topographies adds systematic noise to rs-fMRI, reducing effect sizes and power. Probabilistic map-derived ROIs exhibit higher reliability than group-averaged ones, likely by excluding voxels or grayordinates that demonstrate high network assignment variability across the population (for example, DLPFC and temporoparietal junction). The increased signal-to-noise ratio (SNR) provided by using the probabilistic ROI set allows for additional explanatory reliability when conducting BWAS (Fig. 4c–e). Accounting for individual-specific topography improves reliability with smaller sample sizes and has the potential to increase effect sizes for some investigations (but not all), therefore saving recruitment of potentially hundreds of fewer participants and hundreds of thousands of dollars in MRI scanning costs.

One way to leverage precision mapping in individuals to increase reliability for group studies is to create probabilistic network description region sets. The term ‘precision’ in neuroimaging has been used with many connotations^9,10^ and is often synonymous with collecting hours of connectivity data when signifying reliable personalized network topographies from functional connectivity data. As methods advance, we anticipate the time in the scanner required for precision mapping will reduce. Previous studies focused on interindividual network variability, particularly in connection strength between networks^8,48^. For example, Miranda-Dominguez et al.^48^ have highlighted that the frontoparietal network has personalized network topologies. Sydnor and colleagues^63^ also showed that topological variability relates to complex behaviors. Although fewer publications have highlighted interindividual variability of topography, Dworetsky et al.^22^ and others^23^ have shown that network variability exists across individuals as well. In all of these cases, frontoparietal and transmodal systems exhibit increased variability relative to other unimodal systems, consistent with our findings here.

Probability maps have been used in the structural literature for years; however, there have been limited efforts to produce probabilistic atlases of functional networks, for example, Yeo and colleagues^6^ used a silhouette measure when generating 7 and 17 network solution confidence maps, resembling our ABCD probabilistic maps, but with several notable spatial differences (Supplementary Fig. 18). Others have implemented a group-guided methodology to improve the detection of functional networks by component-based analysis^32,42,64^. These methods typically force participant-specific functional networks to have component weights that are similar to the group representations, which can dilute participant-specific differences in topography. Recent NMF methods decompose the time series into a set of additive parts-based spatial components, yielding a probabilistic parcellation that can be discretized for each participant based on maximal loading to produce individual-specific networks^8,39^. This approach contrasts with TM because we first generate a correlation matrix and then measure the spatial similarity of each grayordinate’s connectivity to a set of networks identified in a group average. By using an approach that leverages the spatial similarity of known networks, we can potentially capture participant-specific functional networks even if participants demonstrate atypical connection strengths seen in neurodevelopmental disorders^65,66^. This methodology is not without limitations, namely that although the functional connectivity of the template was generated from a group of adolescents, networks are initialized by adult group networks.

### Network-specific probabilistic maps have some pros and cons

Structurally informed parcellations (for example, Desikan^67,68^, Destrieux^69^, Melbourne Children’s Regional Infant Brain^70,71^ and HCP atlas^1^) may not consistently reflect underlying functional network topography (Fig. 4b). One major limitation of these parcellations, as well as the Gordon parcellation (333 parcels within ten networks)^5^, is the assumption that a given parcel participates in the same network in all individuals (Fig. 4a). Individual-specific topography confounds this assumption about network assignments. Moreover, atlases that impose network assignments based on gyral-based neuroanatomy likely perpetuate the misconception that identical functions occur at identical locations across individuals, despite obvious interparticipant variation in both gyral anatomy and functional connectivity. Analyses that assume identical network assignments across individuals, based on structurally derived parcellations therefore introduce the following two sources of noise: (1) noise from the misalignment of structural parcellation-to-functional network^72^ and (2) interparticipant network topographic variability, potentially necessitating larger sample size to detect effects reliability.

We found many brain regions that were highly variable in network assignment across the population, namely the DLPFC, and the inferior temporal lobe, regions that have been previously shown to be highly variable in network assignment^22,23^, and transmodal (as opposed to unimodal) in processing^40,63^. We also observed variation in network assignment in the PCC (a region well characterized as being part of the DMN^47,73^) when using single network assignments. Such a finding was illuminated by the integration zones, whereby multiple networks overlapped, suggesting a more complex structure such as a hierarchical structure for networks may be responsible for the noted variation. It should also be noted that the ability to accurately identify either single network or overlapping networks depends on several factors, among which are SNR of the BOLD data^74^, resolution spatial/temporal resolution to reveal network structure^75^ and head motion criteria (Supplementary Fig. 13). Therefore we would expect that subcortical structures, such as the brainstem, would have comparatively worse network consistency relative to the cerebrum or cerebellum (Supplementary Fig. 14).

By considering individual network topographies and/or focusing on areas that are highly consistent across individuals, one may be able to improve power in large-scale studies by limiting the contribution of individual differences to support inferences about the group. The trade-off in the case of the probabilistic regional mappings is that the sparse brain coverage might obscure important information processing that occurs at these omitted variable locations. In predictive analyses, the sparsity of the region set reduces the feature set for prediction; thus, while on the one hand, signals are more reliable relative to traditional region sets, on the other hand, although the unused regions might be less reliable, they could be crucial for maximizing prediction accuracy. Therefore, usage of the MIDB probabilistic ROIs may not suit all situations.

It should be noted that although reliability is improved when using the probabilistic parcellation compared to the Gordon parcellation (Fig. 4d–f), this improvement can only be achieved because each of these cognitive domains makes use of similar network features across the population. However, we want to highlight that the primary goal of this resource is to provide support for investigators to consider individual topographies when asking questions related to the neural architecture supporting learning/memory and executive functions as opposed to having specific hypotheses, per se. With that said, our prior work using NMF shows clear examples of how such information can assist with asking questions about how such information can inform cognitive phenomena^8^.

One important potential usage of probabilistic atlases relates to functional neuronavigation for targeted brain stimulation. Traditionally, noninvasive brain stimulation, such as TMS, relied on anatomical coordinates or task-based fMRI activations for guidance, but recent advancements suggest better outcomes by considering personalized functional connectivity^76^. Recent advances in brain stimulation using TMS have shifted focus from anatomical brain landmarks to personalized fMRI or functional connectivity^15,77^ with the goal of increasing treatment efficacy^15,16^. For instance, targeting the DLPFC based on individualized negative correlations with the subgenual cortex improved antidepressant response^16,62^. However, when neuroimaging data to generate individual maps are lacking or an MRI isn’t available, probabilistic mapping offers a way to optimize targeting across a population.

### Integration zones show hub-like properties

The MIDB Precision Brain Atlas also includes integration zones (IZ) from OMNI mapping that represent overlapping networks. We posit that these integration zones are functionally similar to network hubs^59,78^ (that is, nodes that have a higher degree of connectedness) and likely have a crucial role in relaying information brain networks. Previous investigations^79^ quantified functional connectivity overlap ratio to examine the spatial extent to which each region belongs to a given network. Regions belonging to several networks (for example, posterior parietal and posterior cingulate) closely match those that we identified (Fig. 7)^47^. These zones likely share core features central for cognitive processes like attention and consciousness^80^, showing strong between-group reliability (Fig. 4d–f). This ROI set can offer insights into the mechanisms of information integration and relay and targeted brain stimulation^81^.

Others have examined connectivity between integration zones, or ‘hubs’^59,82^, closely aligning with our integration zones^47^. Conventionally, connector hubs have been conceptualized as specific brain regions that enable network integration, derived from group averages^59,83^. However, it is important to note that despite the spatial variability across participants with respect to the number of overlapping brain networks, the location of integration zones was replicated in an independent group, suggesting that they are not an artifact of group averaging^60^, but are indeed a common feature of network interaction among the population. Comparing BWAS maps between the randomly sampled Gordon parcellation to the integration zones reaffirmed higher reproducibility using integration zones, suggesting their role in the instantiation of complex behaviors (similar to ‘rich club’ areas)^84,85^. It should be noted that the ability to detect grayordinates that overlap with regard to network assignments may be obscured by limited resolution (here 2.4 mm isovoxel). Such resolution, by nature, blurs independent neuronal signals (Supplementary Fig. 12) and might artificially lead to overlapping networks or hubs^86^. Nevertheless, regions with a high density of networks appear to be consistent in the population. Furthermore, neurons that reside at the internetwork boundary likely maintain the boundary through persistent internetwork communication^87^ (Supplementary Fig. 12). Thus, integration zones, while still requiring investigations of their origin, are likely important for the integration of information processing across systems.

### Network maps are a snapshot of topography development

The ABCD study dataset provides a unique opportunity to explore neural networks longitudinally in a set of racially and ethnically diverse young participants, closely representative of the US population^21^. Using participants from the HCP-D dataset, we calculated probabilistic maps for 2-year age bins (8–9, 10–11, 12–13, 14–15, 16–17, 18–19, 20–21 (Fig. 6a)). It should be noted that the comparisons of the MIDB probabilistic regions are against the Gordon parcellation, which was derived in a group of adults. We acknowledge that, despite the Gordon parcellation being used widely in the literature in children, the lack of child-based parcellation generated in a similar manner to the Gordon parcellation is a limitation. However, it has been widely shown that despite brain changes that occur during adolescence, area and network topography with the proper quality control and motion mitigation^37,88^ is largely stable at these age ranges^89,90^. Given this and that the majority of studies within the literature that define the function of these networks have been conducted in adults, the comparison to Gordon atlas, in this context, seems appropriate. Here we provide atlases across multiple ages. Our findings in adults largely replicate, highlighting stability across age ranges. We demonstrate the ROI sets generated from probabilistic maps generated from either adolescents from ABCD or adults from HCP-D (Fig. 3a versus Supplementary Fig. 15a) are nearly identical but also demonstrate nearly identical measures of within-network connectivity. Findings in adults largely replicate, highlighting stability across age ranges. We demonstrate the ROI sets generated from probabilistic maps generated from either adolescents from ABCD or adults from HCP-D (Fig. 3a versus Supplementary Fig. 15a) are nearly identical but also demonstrate nearly identical measures of within-network connectivity. To our knowledge, this is the earliest study to quantify network topography for a sample of this magnitude from adolescence into young adulthood. While we don’t anticipate large changes in network topographies from adolescence to adulthood, there could be refinements around borders^8^. Our resource includes various ages (Supplementary Fig. 15), not just ABCD adolescents, allowing for capturing subtle changes over time in substance abuse, mental health^91,92^, neurocognition^93^, development and environment^94,95^ within the same cohort. As participants age, the MIDB Precision Brain Atlas will provide additional age-specific maps based on ABCD to accompany those provided for HCP-D.

### MIDB open science framework

The MIDB Precision Brain Atlas is an evolving resource, and we invite the scientific community to contribute toward the additional characterization of brain maps. Currently, it offers diverse network brain maps from datasets like ABCD year 1 (ref. ^13^), MSC^9,22^, HCP^22,38^, Yale Low-res^22,96^, Dartmouth Gordon^5,22^ parcellation and HCP-D^36^, with plans to integrate new individual-specific brain mapping techniques as they are developed. The MIDB Precision Brain Atlases will be an evolving repository of processing and analysis tools and parcellations that are overseen by community partners. All individual-specific maps for ABCD will be downloadable through the NDA (https://nda.nih.gov/) or newly associated platforms, which will be updated via the ABCC information page (https://collection3165.readthedocs.io/). All others will be downloadable through the website (per each dataset’s usage agreement). Investigators who wish to share individual-specific maps based on ABCD data can do so via the ABCC (NDA Collection 3165)^13,43^. Contributions to the MIDB Precision Brain Atlas will use the community governance structure (https://bids.neuroimaging.io/governance.html). Briefly, criteria include clear tool descriptions, BIDS format and de-identification. We hope that the thousands of network maps based on multiple validated methodologies and replicable population-level probabilistic topographies we are providing will serve as a new avenue of investigation into adolescent development. Furthermore, the high reliability observed from integration zones merits further investigation as an explanatory source of behavior. As a community-driven atlas, we hope that it fosters systematic studies on network topography and its impact on human cognition and behavior.

## Methods

### Participant information

Analysis of pre-existing neuroimaging data was approved by the University of Minnesota and Oregon Health and Science University Institutional Review boards. Participants consented (or assented when below 18 years old) at their respective collection sites for each study (ABCD^21^: https://abcdstudy.org and HCP-D study^36,99^: https://www.humanconnectome.org/study/hcp-lifespan-development/, MSC^9^: https://openneuro.org/datasets/ds000224/versions/1.0.4) and have agreed to have their anonymized data shared. Participants were recruited under the auspice of the ABCD study to follow brain development and health in a longitudinal manner from 9 to 10 years of age until adolescence. Written informed consent and assent were obtained from a parent or guardian and the child, respectively, to participate in the ABCD study. Behavioral analysis used previously collected behavioral measures including the NIH toolbox tasks, assessments of mental health using Kiddie Schedule for Affective Disorders and Schizophrenia (KSADS) and surveys of substance use, culture and environment from baseline protocols (https://abcdstudy.org/scientists/protocols/).

### ABCD-matched groups

Participants from ABCD were split into three demographically matched cohorts, the ARMS^13,100^. Groups were matched using site, age, sex, ethnicity, grade, highest level of parental education, handedness, combined family income and exposure to anesthesia. Full demographic information for all ABCD cohorts 1, 2 and 3 are described in Extended Data Table 1, with the exception of participants that were excluded. Of these cohorts, participants were excluded either because they were unable to be processed through the DCAN processing pipeline (https://github.com/DCAN-Labs/abcd-hcp-pipeline) described in Methods (typically due to poor image quality) or had fewer than 10 min of resting-state data postmotion correction.

A diagram showing which participants were used to generate probabilistic maps is shown in Supplementary Fig. 1. Brain mapping was performed for all participants with at least 10 min of resting-state data using all three brain mapping methods—IM, TM and NMF. We also used TM and IM separately on concatenated rest and task data using the same network templates used for rs-fMRI data.

### MRI image acquisition

ABCD MRI images were collected from 11,572 participants across 21 sites across the United States of America (Children’s Hospital Los Angeles, University of Colorado Boulder, Florida International University, Laureate Institute for Brain Research, Medical University of South Carolina, Oregon Health and Science University, University of Rochester, SRI International, University of California Los Angeles, University of California San Diego, University of Florida, University of Maryland at Baltimore, University of Michigan, University of Minnesota, University of Pittsburgh Medical Center, University of Utah, University of Vermont, University of Wisconsin-Madison, Virginia Commonwealth University and Washington University in St. Louis)^21^. ABCD participants were aged approximately 9–10 years at the time of collection with ~50% female (see Extended Data Table 1 for additional details). The imaging component of the study was developed by the ABCD Data Analysis and Informatics Center and the ABCD Imaging Acquisition Workgroup. No statistical methods were used to predetermine sample sizes for this manuscript, but the ABCD study is the largest fMRI study conducted in the United States to date and is likely to be sufficiently powered to capture and analyze different patterns of substance use along with many other variables of interest^20,101^ and have been used by other studies to examine brain–behavior relationships^14^. Neuroimaging and behavioral data were collected in accordance with local institutional review boards at each institution.

Sequences were harmonized across Siemens, Philips and GE 3 Tesla (3T) scanners. For further details regarding MRI acquisitions, see refs. ^21,43^. Briefly, participants underwent 25–45 min of prescan task compliance, localizer, 3D T1-weighted MRI (1 mm isotropic, TR = either 2,500 or 6,100 ms, TE = 2–2.9 ms, 8^o^ flip angle, 256 × 256 field of view (FOV)), diffusion-weighted images, 3D T2-weighted MRI (1 mm isotropic, TR = 2,500 or 3,200 ms, TE = 60–565 ms, variable flip angle, 256 × 256 FOV), 1–2 runs of rs-fMRI (2.4 mm isotropic, TR = 800 ms, TE = 30, variable flip angle = 52^o^, 216 × 216 FOV) and a randomized order of monetary incentive delay (MID), stop signal task (SST) and emotional n-back (EN-back) tasks.

Of the original 11,572 participants from the ABCD 2.0 release^20^, participants were divided into discovery (*n* = 5,786) and replication (*n* = 5,786) sets that were matched along the following ten variables: site location, age, sex, ethnicity, grade, highest level of parental education, handedness, combined family income and exposure to anesthesia^93^ (Extended Data Table 1). All resting-state scans were acquired using a gradient-echo, echo-planar imaging sequence (TR = 800 ms, TE = 30 ms, flip angle = 90°, voxel size = 2.4 mm^3^, 60 slices). Head motion was monitored in real time using Framewise Integrated Real-time MRI Monitor (FIRMM) software at Siemens sites^102^. For resting-state scans, participants were instructed to lie still and fixate on a crosshair at the center of their visual field.

All functional MRIs were processed with the publicly available ABCD-BIDS pipeline (https://github.com/DCAN-Labs/abcd-hcp-pipeline), which is a modified version of the HCP processing pipelines^13^. Brain extraction was performed by PreFreesurfer after denoising and bias field correction of the anatomical T1- and/or T2-weighted images. The DCAN-labs processing pipeline applies advanced normalization tools (ANTs) DenoiseImage to improve structural clarity and ANTs N4BiasFeildCorrection (ANTs)^103,104^ to reduce field bias^93^.

### Resting-state time course processing

#### Signal regression

Time courses were corrected using DCAN-BOLDproc^13^. The method for signal regression has been previously described^65^. Briefly, resting-state time courses (using surface registration for cortex and volume registration for subcortical gray matter) were detrended and further processed^105^ using mean whole brain, ventricle and white matter signal as well as displacement on the 6 degrees of freedom, rigid body registration, their derivatives and their squares by regression^106,107^. Finally, time courses were filtered using a first-order Butterworth band pass filter between 9 and 80 mHz backward and forward using MATLAB’s filtfilt function (The MathWorks, v2016-2018x).

The BOLD fMRI volumetric data from the cerebral cortex were constrained to the cortical sheet for surface-based imaging^108^ and combined with volumetric midbrain and hindbrain time courses into a Connectivity Informatics Technology Initiative (CIFTI) format. Once BOLD data were mapped to the sheet, time courses were deformed and resampled to the original surface.

#### Head motion correction

Head movement in the scanner interferes with the ability to identify a grayordinate from one time point to the next, and the movement of a large electrically conductive tissue in a magnet introduces contaminating artifacts from eddy currents. To minimize these effects, we rigorously controlled for head motion by using an FD threshold of 0.2 mm and only using participants with at least 10 min of resting-state data postmotion correction. Movement was calculated by FD in mm using the formula FD_*i*_ = ∣Δ*p*_*i*x_∣ + ∣Δ*p*_*i*y_∣ + ∣Δ*p*_*i*z_∣ + ∣Δ*α*_*i*_∣ + ∣Δ*β*_*i*_∣ + ∣Δ*γ*_*i*_∣, where Δ*p*_*i*x_ is the frame-to-frame change in the x position, *p*: Δ*p*_*i*x_ = *p*_(*i*−1)x_ − *p*_*i*x_, and so forth for the other rigid body parameters (*p*_*i*x_, *p*_*i*y_, *p*_*i*z_, *α*_*i*_, *β*_*i*_, *γ*_*i*_)^109^. Rotational displacements were converted from degrees to millimeters by calculating displacement on the surface of a sphere with a 50 mm radius, which is approximately the mean distance from the cerebral cortex to the center of the head. Frames were removed if their total relative movement in any direction (FD) was greater than 0.2 mm relative to the previous frame or if they were contained within a segment of five contiguous frames that violated the threshold.

For the remaining frames, the s.d. was calculated across all grayordinates to remove potential artifacts. Frames that had outliers in the s.d. of the bold signal were removed using the median absolute deviation method in MATLAB and Statistics Toolbox Release 2016b-2022b (The MathWorks). In time courses containing more than 10 min of resting-state data, frames were randomly sampled to generate correlation matrices using exactly 10 min of fs-MRI data. Of the 11,572 participants enrolled, 10,079 in groups 1 and 2 had usable structural and functional MRI collected, and of these based on our movement/signal criteria, approximately 3,973 (~40%) children were excluded based on excessive movement in the scanner during resting-state scans, resulting in 2,995 (group 1) and 3,111 (group 2) participants. During task-based scans, we were able to retain many more usable frames at the FD criteria (group 1, *n* = 4,699 and group 2, *n* = 4732), excluding only 607 participants (6%).

#### IM community detection method

The community detection method using the graph theory-based algorithm IM has been previously described^7,9^. The same correlation matrices that were used in the TM processes were used to detect networks using IM. Briefly, vertices/voxels within 30 mm of each other were set to zero in the matrix to avoid biasing network membership for nearby connections that had undergone spatial smoothing. The resulting correlation matrix was then thresholded at a range of density thresholds (0.3%, 0.4%, 0.5%, 1%, 1.5%, 2.0%, 2.5% and 3.0%) and each one was used as an input for IM. For instances where IM was implemented on combined cortical and subcortical data (data shown in Fig. 3c and the average group matrix shown in Supplementary Fig. 6), we extended the range of density thresholds to include 4% and 5%. IM calculates the network assignment based on an optimized code length using a flow-based method^31,41^. Networks that are computed in the group average are labeled based on similar patterns of activation observed in the scientific literature^9,23^. Small networks with 400 or fewer grayordinates were defined as ‘unassigned’.

Networks identified in each individual were then labeled based on the Jaccard similarity index to a network observed in the group average; however, often individuals will retain new networks that are not observed in group averaging, and these remain unlabeled. The list of networks included is the DMN, the visual network (Vis), the frontal-parietal network (FPN), the dorsal attention network (DAN), the ventral attention network (VAN), the salience network (Sal), the cingulo-opercular network (CO), the sensorimotor dorsal network (SMd), the sensorimotor lateral network (SMl), the auditory network (Aud), the anterior medial temporal network, the posterior medial temporal network (post-MTL), parieto-occipital network (PON) and the parietal medial network (PMN)^9,22^. In each participant and in the average, a ‘consensus’ network assignment was determined across the various thresholds, by giving each node the assignment it had at the sparsest possible threshold at which it was successfully assigned to one of the known group networks (Supplementary Fig. 2). Contiguous network clusters that were smaller than 30 grayordinates were removed and merged into neighboring networks, with the largest networks given priority.

Similar to how a letter in the United States can be addressed to a house with a two-level description state/province, then city, brain network organization can be described using a two-level system of networks and nodes, respectively. IM is a network-describing algorithm that tries to minimize the number of bits (using Huffman coding) necessary to describe the whole network^31,110^. For example, would it require fewer bits to describe the whole brain with few networks containing many nodes, or many networks with fewer nodes? IM uses a random walk algorithm that uses connection weights to determine the minimum descriptor code length necessary to describe the structure. Notably, while the solution provides modules, it is not designed to maximize modularity. As others have done previously^9^, we thresholded the correlation matrix to the top x% of connections (or edges) because of the computational limitations of using a full set of 4.1 billion connections as descriptors in the map equation. We thresholded the connectivity matrix at a threshold of 0.3%, 0.4%, 0.5%, 1%, 1.5%, 2%, 2.5% and 3%. These thresholds were chosen to scale the number of edges in proportion to edge densities that have been used previously^9^.

To generate a consensus across multiple edge densities, we implemented a methodology developed by Gordon et al. ^9^. Briefly, after IM detected communities for each participant, putative network assignments were then assigned to each participant’s communities by matching them at each threshold to the independent group networks from WashU (*n* = 120). To do this, for each individual, at each density threshold, the spatial overlap of each unknown community was compared to each one of the independent group networks separately using the Jaccard similarity index. The unknown community was then assigned that network identity to which it had the highest Jaccard similarity index. If the Jaccard Index was less than 0.1, the community remained unassigned, so as to avoid assigning communities to known networks based on only a few vertices. Assignments were first made with the large, well-known networks (default, lateral visual, motor, frontoparietal, cingulo-opercular and dorsal attention) and then to the smaller, less well-known networks (ventral attention, salience, parietal memory, parieto-occipital, temporal pole, medial temporal). In each individual, a ‘consensus’ network assignment was created by giving each grayordinate the assignment it had at the sparsest threshold at which it was successfully assigned to one of the canonical group networks.

#### TM method

Multiple versions of the time series were used depending on the analysis—either exactly 10 min of randomly sampled frames, all available frames below the FD threshold, or concatenated rest and task data in the following order: rest, MID, n-back and SST (provided that the participant had an available scan for the task). To generate the templates, IM community detection was performed at several tie densities (for full details of average networks, see refs. ^9,23^) on an average connectivity matrix (*n* = 120 participants) using a two-level solution. This yielded 14 networks that include the DMN, the Vis, the FPN, the DAN, the VAN, the Sal, the CO, the SMd, the SMl, the Aud, the temporal pole network (Tpole), the MTL, the PON and the PMN. Sensory and motor systems were combined due to the coupled nature of activation. Despite high reproducibility in resting-state functional connectivity, the extent to which these networks are activated on a neuronal time scale is unclear. However, recent work discussed in ref. ^58^ suggests that the contribution of short-term dynamic changes (for example, from task-based states) to variation in brain organization is quite modest relative to resting-state organization.

A graphical description of the TM method is shown in Supplementary Fig. 3. Gordon et al. ^9^ generated single network assignments using IM on a group average dense connectivity matrix from a cohort of 210 adults. The parcellation was used to anatomically define networks for each participant and create seed-based correlations for each network in all participants in the template group (*n* = 164 ABCD-group 3 participants). Seed-based correlation maps were averaged across the participants in the template group for each network separately (Supplementary Fig. 3a). Each template was then thresholded to correlation values >*z* score = 1 (~top 15.9% of connections). To perform TM in the group 3 participants, we first generated whole-brain connectivity matrices. Here we show an example of the whole-brain connectivity of a grayordinate within the PCC. We then thresholded the connectivity for each grayordinate in the same manner as the template and calculated an *η*^2^ value for each network. Each grayordinate is then assigned to the network based on the maximum *η*^2^ value (Supplementary Fig. 3b).

To generate an independent template, we conducted a seed-based correlation (using an average time series correlated to all the grayordinates) for all networks. Seed-based correlations were generated using the dense time series from each template participant that was smoothed with a within-frame spatial Gaussian smoothing kernel of 2.55 mm using each participant’s own mid-thickness surfaces (extracted from the Surf stage of Freesurfer). The resulting networks were converted to a dlabel CIFTI file and applied to the smooth dense series to generate an average time series for each network. We then correlated the time series of the seed with the time series of all other grayordinates. The seed and remaining time series were motion-censored using an FD of 0.2 mm, and outliers in the BOLD signal were removed using the median absolute deviation in the remaining frames using the motion-censoring method outlined above.

Seed-based correlation values were averaged across all the participants in the template group (*n* = 164, 9–10-year-olds), resulting in a vector (91,282 × 1) of average correlation values for each network correlated with each grayordinate. Each network vector was averaged independently across participants in the template group to generate seed-based templates for each network. We then thresholded each network template at *z* ≥ 1.

To generate precision maps for each participant in ABCD-group 1 and ABCD-group 2, we examined the whole-brain connectivity for each grayordinate by correlating the dense time series against all other grayordinates. For each participant in each test group (group 1, *n* = ~5,000 and group 2, *n* = ~5,000), we generated a Pearson correlation matrix (91,282 × 91,282 grayordinates) for each connection using the dense time series using the Connectome Workbench command ‘-cifti-correlate’ (https://www.humanconnectome.org/software/connectome-workbench). Time series were then motion-censored (‘Head motion correction’) to reduce artifacts induced by head motion.

Because connectivity matrices were generated including subcortical brain regions, the correlation matrix was *z*-scored separately for each hemisphere, the subcortical region, and the connections between the cortex and the subcortex. This allowed for the normalization of connectivity between subcortex and cortex where there is the potential for a decreased SNR in the subcortex. We thresholded the whole-brain connectivity for each grayordinate to only include correlated grayordinates with *z-*score values greater than or equal to one. This resulted in a vector of whole-brain connectivity for each grayordinate that only includes grayordinates that are strongly correlated to a given network template. We then calculated an *η*^2^ value between the remaining grayordinates and each of the network templates seen in Supplementary Fig. 4. The grayordinate is assigned to whichever network with the maximum *η*^2^ value.

#### OMNI mapping method

To generate overlapping networks for each participant, rather than assigning the grayordinate to the network with the maximum *η*^2^ value, we used a data-driven approach to assign multiple networks to each grayordinate. For each network, we plotted the distribution of *η*^2^ values (Fig. 5c). The connectivity for each network demonstrates a characteristic skewed bimodal distribution. The distribution for *η*^2^ values was distributed into 10,000 bins and fitted with a cubic spline. The distribution was then smoothed using a Savitzky–Golay filter using a 2,000 data point window within MATLAB (The MathWorks, v2016-2022x). We calculated the local minimum of the bimodal distribution by taking the derivative of the smoothed data between 4,000 and 7,000 bins. We then used this local minimum as the threshold for whether or not a grayordinate would be labeled with this network, where grayordinates above this threshold would receive the network assignment. Grayordinates that had an *η*^2^ value higher than the threshold were assigned to those networks (Fig. 5d). Notably, unlike the winner-take-all approach, this method does not require an assigned network at each grayordinate, whereby grayordinates that do pass this thresholding and are not preferentially linked to any given network will go unassigned. In Supplementary Fig. 7a, an example of overlapping networks using OMNI mapping is shown for an ABCD participant with 10 min of low-motion resting-state data. Because each grayordinate can belong to multiple networks, we used the ten ABCD participants mentioned above to calculate NMI independently for each network (Supplementary Fig. 7). Networks that have larger topographical variability among the population (for example, the frontoparietal network) are those that had larger difference in NMI between intraparticipant split halves compared to the null distribution, indicating topographical specificity (Supplementary Fig. 7b, yellow distribution versus black distribution). Probabilistic mapping of overlapping networks for each group revealed that these probabilistic maps were reliable (see Fig. 6 and Supplementary Fig. 11 for all the network maps).

OMNI mapping allowed us to identify regions of network overlap and integration zones. One might be tempted to interpret the observed integration zones as a byproduct of volumetric averaging due to the limited volumetric resolution of rs-fMRI (Supplementary Fig. 12d–f). rs-MRI is generally collected with 3–4 mm resolution to optimize the SNR when using a 3T scanner^111^. There is evidence to suggest that smaller voxels produce a higher SNR and stronger BOLD effects at high fields such as 7T, which, at least within the motor system, can significantly affect the estimate of intervoxel correlation^112^. Newton and colleagues^112^ demonstrated that BOLD imaging at very high spatial resolution (1 × 1 × 2 mm) allows for improved functional connectivity analyses, allowing them to distinguish the intricacies of the sensorimotor network (as defined by a finger tapping task compared to rest) in resting-state functional connectivity maps, which may be attributable to due to decreased partial volume averaging. Any voxel size larger than a single hemodynamic unit (a neuron, corresponding capillaries and supporting astrocytes) is going to be susceptible to volumetric averaging. However, while volumetric averaging resulting from our collection resolution (2.4 × 2.4 × 2.4 mm) does occur, there are several reasons why it is still likely that neurons residing at the boundaries between networks are important for integration.

First, integration zones appear to be in generally similar locations across the population. If volumetric averaging contributed to the overlapping integration that we’ve observed, then we would expect them to exist indiscriminately near the boundaries of all networks. Instead, what we observe is that integration zones are present at relatively similar network intersections across participants. Second, the location of the integration zones closely corresponds to hubs with regions that are either highly metabolically active^47^, relay information between nodes^85^ or process multimodal information^113,114^, which supports the hypothesis that these regions are likely integrating information from multiple networks.

Furthermore, discrete network boundaries such as those shown in Supplementary Fig. 12b do not preclude neurons at the interface boundary from communicating with one another. On the contrary, they reinforce the boundary through persistent internetwork communication. Techniques that implement boundary mapping are predicated on the observation that resting-state functional connectivity patterns can abruptly change from one cortical region to an adjacent cortical region, which often reflects the abrupt changes in cytoarchitectonics in the cortex in nonhuman primates^5,115^. Few studies have examined cross-network communication at the resolution necessary to capture the nuances of integration between networks. However, in one study of adjacent brain regions, Carmichael and Price identified two distinct networks within the macaque orbital and medial prefrontal cortex using retrograde and anterograde tracers^87^. Although the regions’ networks were clearly distinct, they were highly interconnected at the boundary region between them^87,116^.

#### Probabilistic maps

Probabilistic maps were generated separately for each group, method and network. Probabilistic maps were generated by calculating the probability that a grayordinate was assigned to a given network using all the participants within the group. The TM ROI set was generated by converting clusters produced by thresholding the probability maps at 0.8 (excluding clusters smaller than 30 grayordinates), converting them to dlabel files (dlabel.nii) and combining ROIs into one combined probabilistic parcellation. Probabilistic dlabel files are available for the combined networks and each network separately from the MiDB Precision Brain Atlas webpage: http://neuroatlas.org. We performed an additional analysis with participants separated by site. When we correlated probabilistic maps between sites, we observed that probabilistic maps were nearly identical (Supplementary Fig. 16).

#### NMF community detection method

We implemented a community detection technique used previously^8^ to decompose non-negative participant-specific functional networks using their corresponding concatenated rest + task dense time series in a constrained manner using three regularized terms^32,46^. Briefly, a voxel-wise group sparsity regularization term was first used to ensure that a group consensus was used as a prior using group 3. Second, the spatial locality regularization term was used to ensure that functional coherent voxels are encouraged to reside in the same functional network. Finally, a within-participants regularization term was used to eliminate redundant functional networks^8,32^. The weights from the consensus were then applied to each of the time series for participants in ABCD-group 1 and ABCD-group 2.

#### Analysis of minutes necessary for reliable communities using split halves

We calculated the similarity between split halves for an individual by splitting the resting-state time series in half and generating a correlation matrix of all grayordinates from each half using exactly 10 min of randomly sampled frames. Each correlation matrix was used as an input to both the TM algorithm and IM algorithm to generate networks for each half (‘TM method’). We then calculated the NMI between halves (https://github.com/MidnightScanClub/MSCcodebase). To create the null distribution, we calculated the NMI between an individual participant’s half and all other halves in the group set for all participants. The difference between the test (self) and null (other) distributions was assessed using an independent two-sample *t* test with unequal variance.

#### Brain–behavior associations using subset reliability

To assess the reliability of a probabilistic parcellation schema, we conducted a split-group subset reliability association analysis. We randomly sampled participants from group 1 at discrete sample sizes and correlated each corresponding element of the matrix to measure reliability against the participants’ behavioral measures. For each analysis, we quantified the correlation between each participant’s behavioral measure and (1) the Gordon connectivity matrix, (2) the probabilistic parcellation connectivity matrix or (3) the integrative zone. The resultant correlation matrix for each subset was then correlated to the correlation matrix made from all the participants in group 2. To calculate a nonlinear regression estimate across sample sizes, we then fitted a curve through the data points using an exponential rise-to-maximum single 3-parameter estimate (SigmaPlot 12.5 (Systat Software)) with the following equation:

$$y={y}_{0}+a\times (1-{\mathrm{e}}^{-{bx}})$$

where *y* is the correlation, *y*_0_ is the *y*-intercept, *a* is a scaling parameter, *b* is the rate of rise to maximum and *x* is the number of participants. All regression parameter fits were significant (*P* < 0.0001) and were highly correlated with the data (PC1 Gordon: *r*^2^ = 0.8045, TM: 0.8540; PC2: Gordon = 0.7181, TM = 0.6260; PC3: Gordon = 0.8086, TM = 0.6999). The coefficients for the curves for each model are provided in Supplementary Table 5. To ensure that the increase in intergroup reproducibility observed with the integration zones was not simply due to the reduced number of ROIs, we conducted an additional subset reliability analysis, where we randomly sampled 80 ROIs from the Gordon parcellation (the same number of ROIs in the TM region set) at each of the various participant subsets (Fig. 4d–f). Neural network graphs can be mapped at multiple scales, and the reliability when resolving network assignments at those scales can vary depending on the dataset and the community detection algorithm^117^. However, thresholding by population-level network probabilities ultimately yields different numbers of ROIs, but more importantly, this ROI set is not based on thresholding a graph (as is done for IM), so assumptions about scale/resolution may not apply to this region set. Because the number of regions differs between the MIDB probabilistic parcellation and the Gordon parcellation, the within-network connectivity was not normally distributed, so for every network, equal variance was not assumed for these statistical tests.

#### Data requirements for network specificity

An open question in the field of neuroimaging is ‘What amount of resting-state data is required to draw reliable conclusions about an individual’s connectome?’. Some estimates examining split-half reliability of connectivity matrices have demonstrated that upwards of 30 min of low-motion BOLD data are necessary^11^. We performed a split-half reliability analysis for network maps generated in ten participants from the MSC dataset, who underwent 5 h of rs-fMRI (in addition to task collection)^9,118^. We split the resting-state scans into interleaved halves, generated networks from each half as described in Methods and calculated the NMI between networks generated from halves of the within versus between participants (identical to the analysis shown in Fig. 3b). As with the ABCD dataset, the NMI of networks generated from the same MSC participants was significantly higher than networks from different participants. In Supplementary Fig. 5, the range of same-participant NMIs is shown in a blue box (0.527–0.648) and the range of null NMI (from comparing different participants) is shown as a gray box (0.314–0.378). Interestingly, the average intraparticipant NMI from MSC participants was higher than ABCD participants (MSC: 0.584 versus ABCD: 0.4214), suggesting that random sampling from longer/multiple sessions may produce more reliable network maps.

In addition to the split halves analysis, we also compared the similarity of networks generated from the second half of a participant’s data (average of 71.28 ± 37.82 min, FD = 0.2) versus networks generated from discrete time intervals (1, 2, 3, 4, 5, 10, 15 and 20 min, 10 times each) randomly sampled from the first half (average of 73.12 ± 43.72 min, FD = 0.2). The NMI between network maps generated from each interval compared to the second half rapidly increased as correlation matrices contained more time points up to 5–10 min, then began to plateau. Only 2 min of resting-state data were needed to generate intraparticipant network maps with greater similarity than to the other participants in the group (1 min: *t*(9.68) = −3.37, *P* = 0.0074; 2 min: *t*(9.33) = 8.919, *P* = 7.211 × 10^−6^; 3 min: *t*(9.50) = 17.10, *P* = 1.858 × 10^−8^; 4 min: *t*(9.26) = 15.200, *P* = 7.33 × 10^−8^; 5 min: *t*(9.27) = 17.554, *P* = 1.978 × 10^−8^; 10 min: *t*(9.19) = 18.201, *P* = 1.6055 × 10^−8^; 15 min: *t*(9.20) = 20.33, *P* = 5.839 × 10^−9^; 20 min: *t*(9.15) = 18.603, *P* = 1.3864 × 10^−8^, two-tailed, unequal variance assumed; Supplementary Fig. 5b). For most MSC participants, only 10 min of data were required to generate network maps with NMI values that fell within the range of the expected maximum NMI. The probabilistic ROIs generated from the ABCD participants used 10 min of randomly sampled data; however, sampling from longer data collections, such as the ones collected in the MSC dataset, has the potential to artificially inflate the similarity between halves due to the reduced influence of autocorrelation in the time series. Therefore, in addition, we sampled 10 min of continuous low-motion data that were motion-censored in an identical manner as described in Methods. The only exception was that the time intervals were not randomly sampled throughout the collection, but rather a random low-motion frame was selected, and the amount of subsequent low-motion frames corresponding with each time interval was used to generate a correlation matrix. Network maps were then produced by TM in the same manner as described in the main text. On comparing Supplementary Fig. 5a with Supplementary Fig. 5b, we observed that the randomly sampled frames generated more similar maps between halves (as evidenced by the increase in NMI) than the continuously sampled data. The NMI for the group continuously sampled data is significantly greater than the null for time intervals longer than 5 min; however, specificity is indicated when data points are no longer in the gray-shaded regions (Supplementary Fig. 5). All network maps using continuous data for all MSC participants were outside the gray region after using 10 min of continuously sampled data, suggesting that sampling from longer time intervals does improve reliability that others have shown^11^.

It is unknown whether adolescent brains demonstrate similar reliability in network topography to that of adult brains. Therefore, we aimed to collect additional data in an adolescent sample. Because the ABCD study did not collect a sufficiently long duration of resting-state data to examine reliability in a similar manner to that of the MSC dataset (Supplementary Fig. 5), we collected additional long-duration rs-fMRI data in a group of child/adolescents at the Masonic Institute for the Developing Brain (MIDB subpopulation cohort), to test reliability in a similar way as done in the adult cohort. We examined the reliability of networks in 5 and 9–10-year-old children by splitting dense time series data in half. Then we randomly sampled minutes (average total time 143.0 ± 28.66 min) from the first half and compared them to the second half. In Supplementary Fig. 19, we compare split-half reliability in cortical networks in adolescents (left) to adult networks (right). The pattern of split-half reliability is mostly similar to what we observed in adults, albeit actually a little bit better. At 5 min of randomly sampled data, roughly half of the participants are within the range of maximum reliability (blue region), and by 10 min nearly all participants are in this range. Using a nonlinear curve-fit (3-parameter exponential rise-to-maximum function: *f*(*y*0) + *α* × (1 − *βx*)), we observed that the curvature is similar between these datasets (adolescent, *β* = 0.609 and adult, *β* = 0.6153). It should be noted that there are a few differences in interpreting the reliability compared to the MSC, notably that the adolescent participants were collected with a multi-band, multi-echo sequence^119,120^, and processed using an updated version of fMRIprep pipeline^121^ that our lab has assisted in building, as the abcd-hcp-pipeline has not been modified to handle multi-echo data.

#### Network topography group replication in matched samples

In addition to brain mapping on an individual basis, we also created network maps from average dense connectivity matrices for groups 1 and 2 to show replication across independent samples and across methods (Supplementary Fig. 6). IM and TM brain mapping methods were applied to identical connectivity matrices generated from matched groups. To highlight reproducibility we measured the amount of replication using an average dense connectivity matrix generated from all participants within each independent group (Supplementary Fig. 6; group 1 and group 2; see Supplementary Table 1 for demographic details). We calculated the NMI between groups and between methods for group-specific networks. The NMI between group 1 and group 2 was relatively high for each method (TM: 0.9110; IM: 0.7893), suggesting that each method provides robustness to replication. We also used NMI to compare the similarity of networks generated from TM versus IM for each group (Supplementary Fig. 6). Between methods, groups generally display similar topographies as evidenced by high NMI values (group 1, 0.4798 and group 2, 0.4762), relative to the null comparison between participants (Fig. 1). IM (upper row) and TM (lower row) produced relatively high replication as evidenced by split-group NMI. Insets show that the network labels identified for subcortical regions and cerebellum are markedly similar across groups as well.

### Reporting summary

Further information on research design is available in the Nature Portfolio Reporting Summary linked to this article.

## Online content

Any methods, additional references, Nature Portfolio reporting summaries, source data, extended data, supplementary information, acknowledgements, peer review information; details of author contributions and competing interests; and statements of data and code availability are available at 10.1038/s41593-024-01596-5.

### Supplementary information

Supplementary Information Supplementary Figs. 1–19 and Tables 1–7.

Reporting Summary

## Figures and Tables

**Extended Data Table 1:**
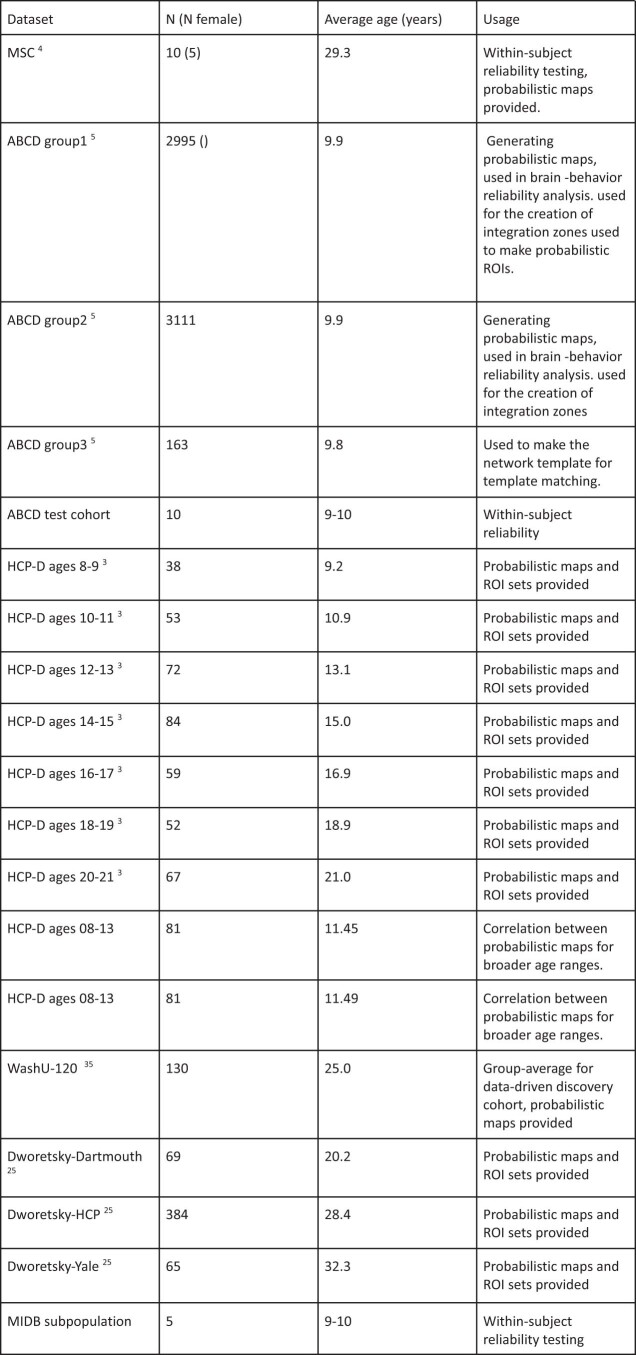
Neuroimaging datasets and their respective usage in this paper

This table describes the source of the source neuroimaging cohorts described in the manuscript, the sample size, N, the average age of participants, and how the cohort is used with each analysis.

**Extended Data Table 2:**
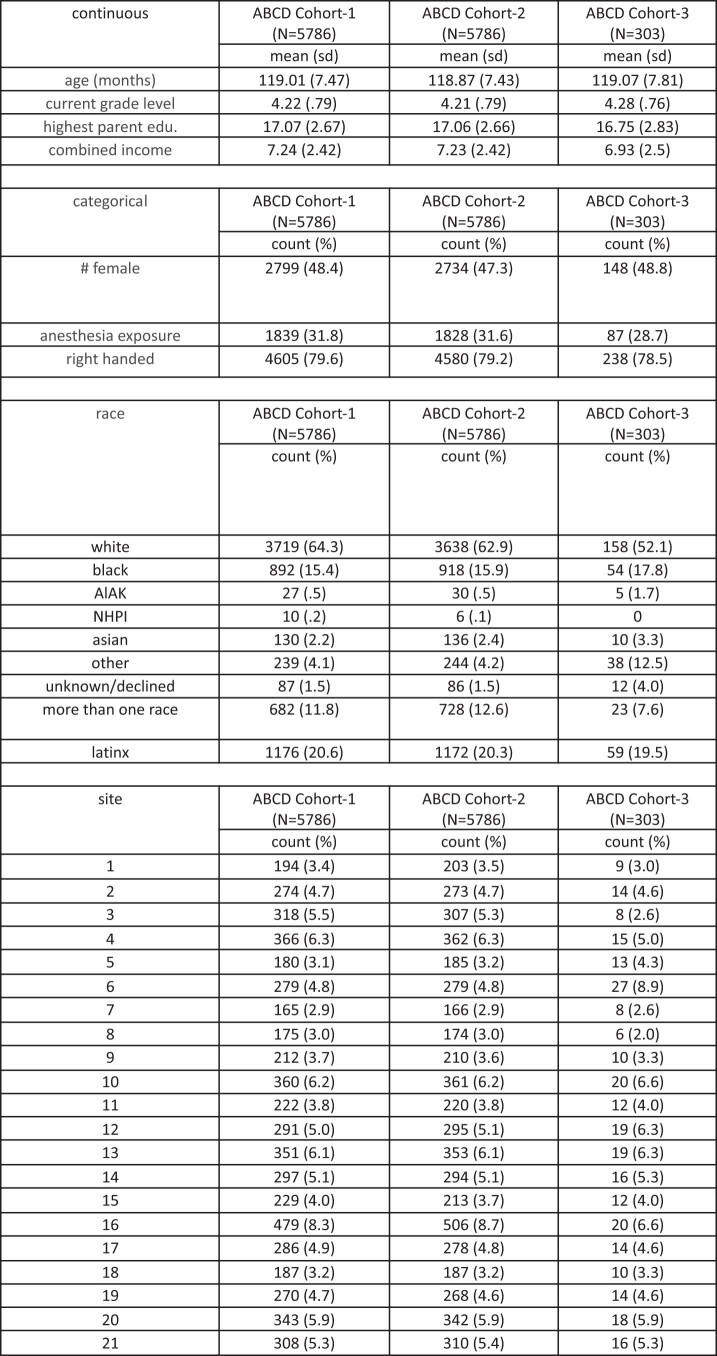
Demographics of all ABCD participants for year 1

This table provides demographic information summarizing the three ABCD cohorts that were collected (full sample).

**Extended Data Table 3:**
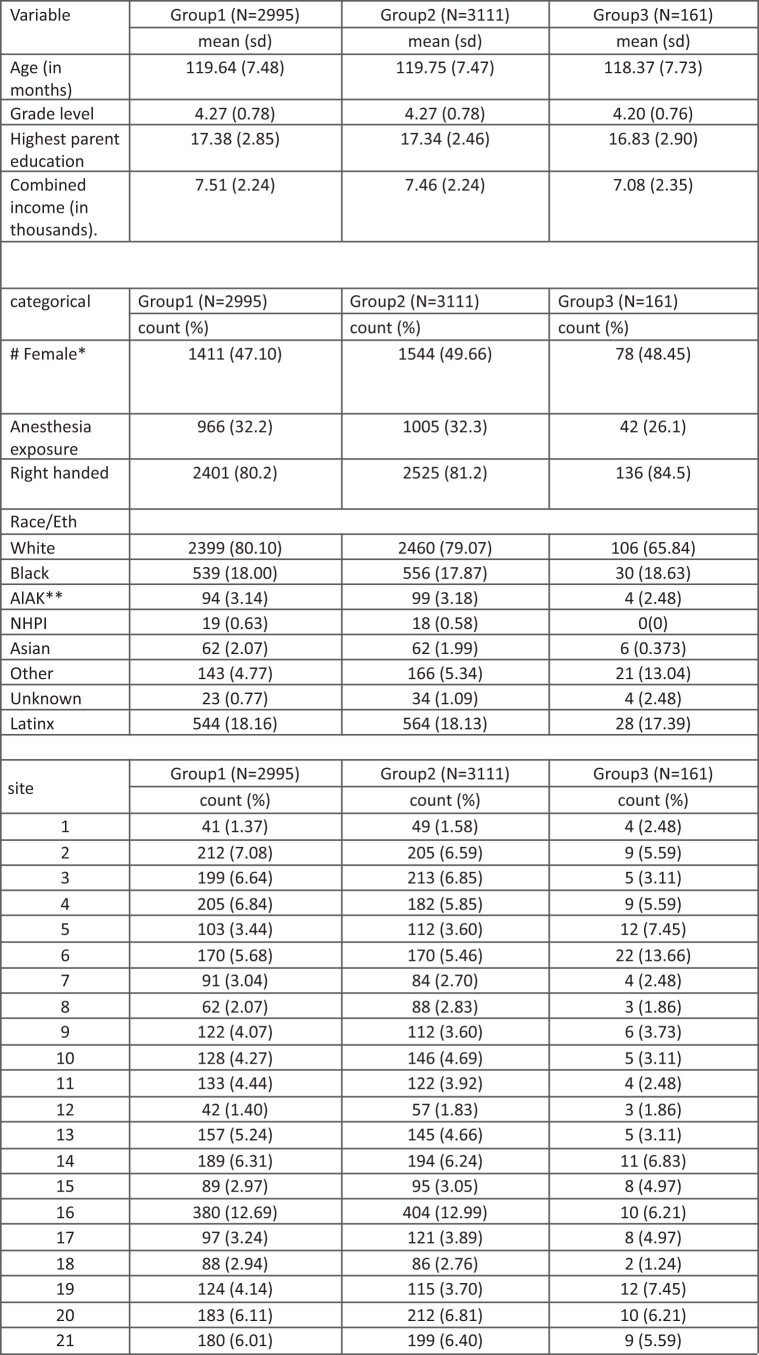
Group demographics table—participants with at least 10 min of resting-state data

This table provides demographic information summarizing the three ABCD groups that had at least 10 min of resting-state data.

## Data Availability

ABCD neuroimaging data and behavioral data constitute the minimum dataset used to generate findings in this study and are currently available from the NDA upon approval with a data use agreement (https://nda.nih.gov/). The data will also be available on any future release platform. Updates will be announced on the ABCC information website (https://collection3165.readthedocs.io/). All individual-specific maps for ABCD will be downloadable via these platforms as well (pending approval). Investigators who wish to share individual-specific maps based on ABCD data can do so via the ABCC (instructions provided on the ABCC information page and https://midbatlas.io/)^13,43^. Probabilistic maps from each network are available at https://midbatlas.io/. The MSC data are publically available at https://openneuro.org/datasets/ds000224. Individual-specific maps and connectivity maps are available at https://nda.nih.gov/edit_collection.html?id=3165. HCP and HCP-D data are available at https://www.humanconnectome.org/. Data associated with the WashU-120 are available at https://openneuro.org/datasets/ds000243/versions/00001.

## Code availability

Custom computer code that was used to process the neuroimaging data using surface-based analysis can be found here and is publicly available at https://github.com/DCAN-Labs/abcd-hcp-pipeline (v0.1.0). Code to generate connectivity matrices (v1.0) from time series data can be found at https://github.com/DCAN-Labs/cifti-connectivity and is publicly available. Custom code to generate individual-specific networks and probabilistic maps can be found at https://github.com/DCAN-Labs/compare_matrices_to_assign_networks (v1.0) and is publicly available. IM community detection code is available at www.mapequation.org (v1.4 was used in this manuscript). Additional tools used to work with CIFTI files that were used to support the findings of this study were as follows: MATLAB 2019b–2022b (MathWorks, https://www.mathworks.com/), Connectome Workbench^122^ (v1.4.2–1.5.0; http://www.humanconnectome.org/software/connectome-workbench), Freesurfer^123^ (v6.0–7.4.1; https://surfer.nmr.mgh.harvard.edu/), FSL^124^ 5.0 and above (https://fsl.fmrib.ox.ac.uk/fsl/fslwiki), code from the MSC (v1.0) (ref. ^9^; https://www.openfmri.org/dataset/ds000224/), MIDB ROI tool (R.J.M.H. and J. Fair: https://midbatlas.io/), ABCC for matched groups v1.0.0 by ref. ^13^, https://www.biorxiv.org/content/10.1101/2021.07.09.451638v1. fMRIPrep v23.0.0–23.1.4. (https://fmriprep.org/) was used for processing multi-band, multi-echo fMRI data. ARMS software code (v1.0) that was used to generate matched groups can be found here: https://osf.io/7xn4f/.
