# Supplementary material for: A precision functional atlas of personalized network topography and probabilities
Source: Nat Neurosci. 2024 Mar 26;27(5):1000–13. doi: 10.1038/s41593-024-01596-5 (PMC11089006; doi:10.1038/s41593-024-01596-5)
Supplement: Supplementary file 1 — Supplementary Figs. 1–19 and Tables 1–7. [file 41593_2024_1596_MOESM1_ESM.pdf]

nature neuroscience

Resource https://doi.org/10.1038/s41593-024-01596-5

A precision functional atlas of personalized network topography and probabilities

In the format provided by the authors and unedited

SUPPLEMENTARY INFORMATION

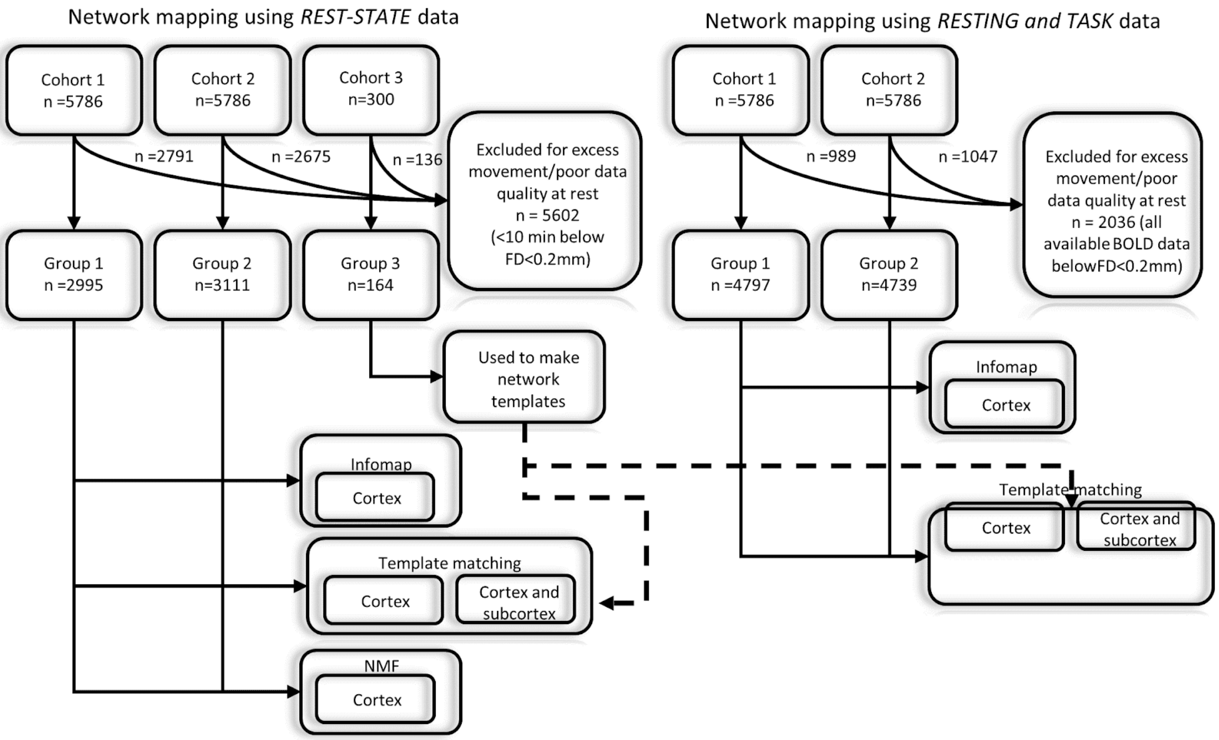

Supplementary Figure S1) *Participant cohorts and types of community detection implemented. Participants were placed into matched cohorts (see demographics in Extended Data Table 2 for additional demographic information of all the subjects).*

Additional Community Detection Methodologies

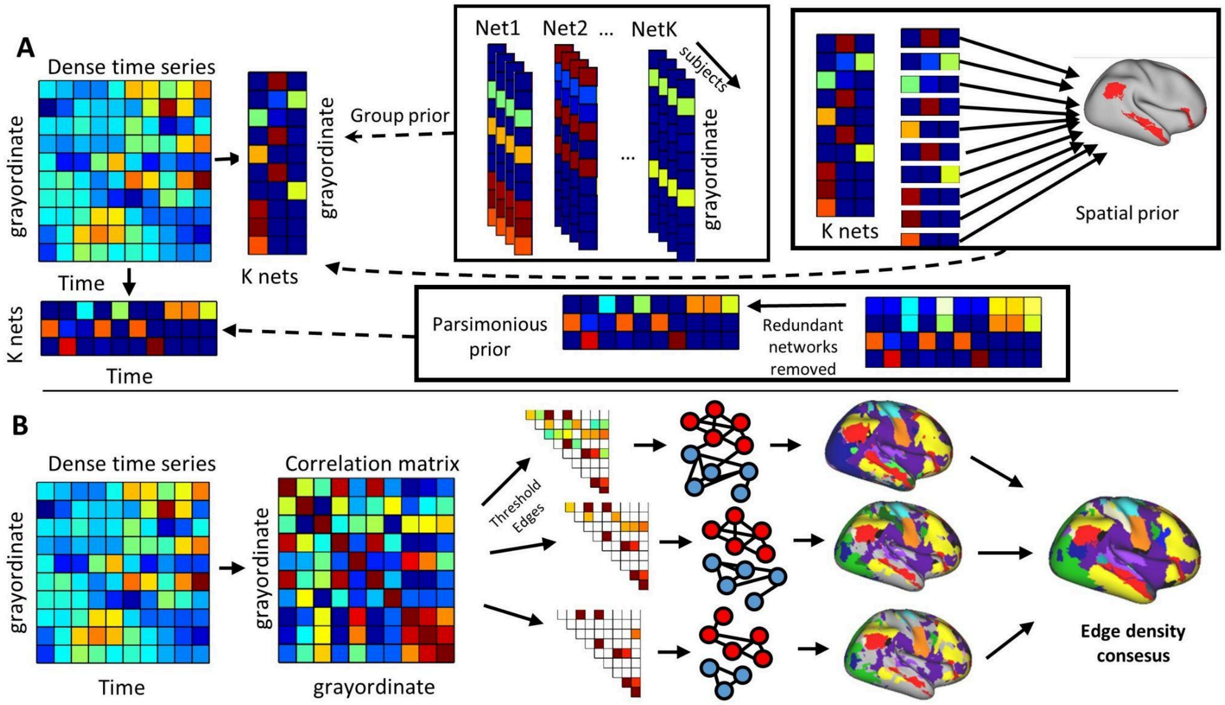

Supplementary Figure S2) NMF and Infomap methods of community detection. A) Non-negative matrix factorization was conducted on the dense time series using 3 priors: A group consensus prior which encourages subjects to have a similar number of networks, and a spatial prior which serves to constrain the spatial topology of a given network, a parsimonious prior, which removed redundant networks for each subject. B) When Infomap community detection was implemented, we generated a correlation matrix using motion-censored dense time series data (see methods in main text) in an identical manner to the template matching procedure. Each upper triangle of the correlation matrix was then thresholded to various (0.3, 0.4, 0.5, 1.0, 1.5, 2.0, 2.5, 3.0, 3.5, 4.0, 4.5, 5.0) top percentages of the connections. Those connections were then used as the input for Infomap. Infomap uses a random walk to minimize bit-wise code length necessary to describe the whole system structure. The final network labels were then determined by generating a consensus across thresholds, then comparing the Jaccard index of the spatial arrangement of grayordinates from the detected network with those found in the group (See methods).

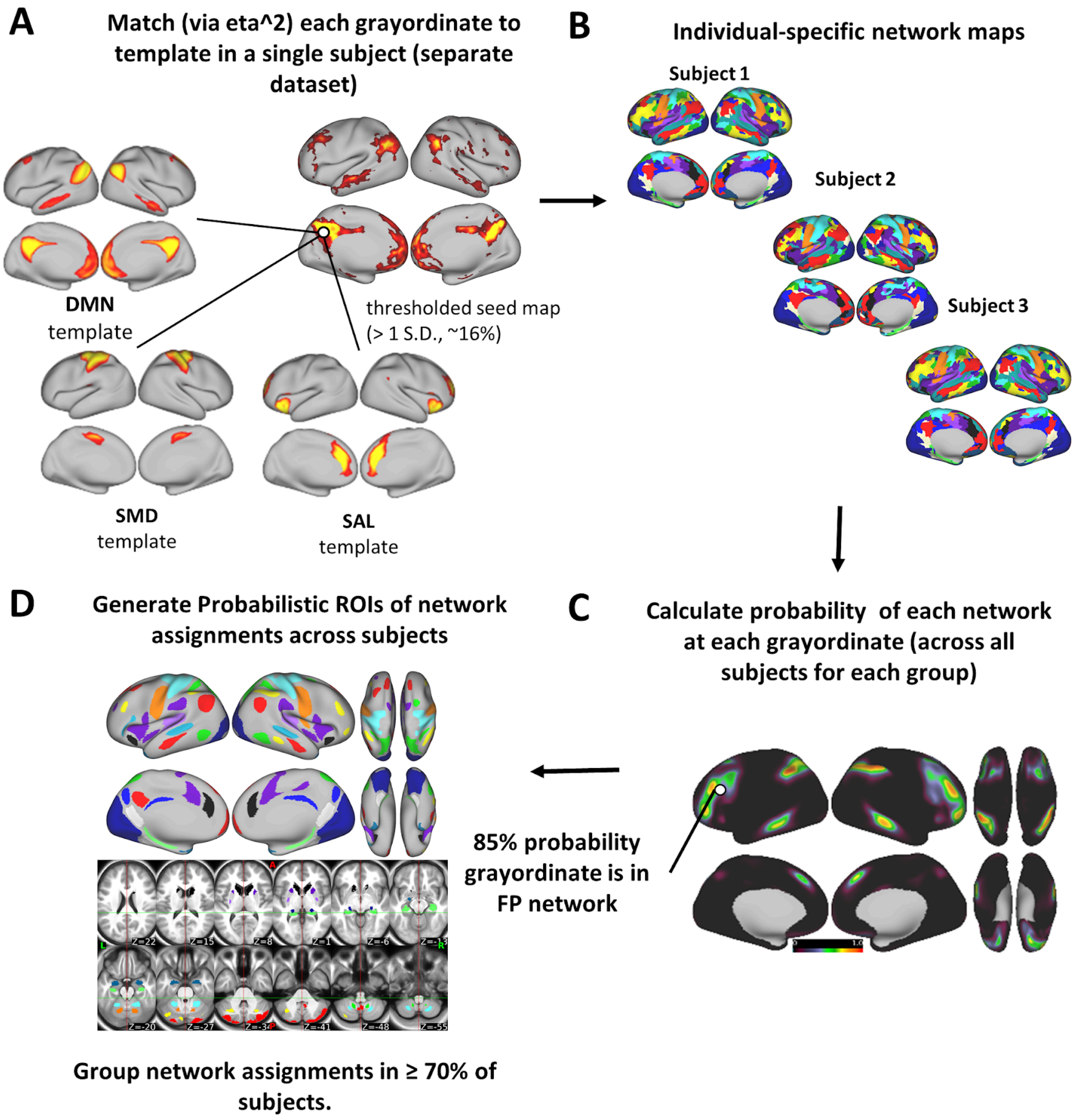

Supplementary Figure S3) Template matching method. *A) For the test subjects, we then generated* whole brain connectivity matrices. Each row of the connectivity matrix was compared with each of the network templates. (only DMN, SMD, and SAL are shown here for visualization purposes). Here, we show an example of connectivity to a grayordinate within the posterior cingulate cortex (PCC), whose connectivity resembles the default mode network. Each template was thresholded to correlation values >Zscore =1 (~top 15.9% of connections). We then thresholded the connectivity for each grayordinate in the same manner as the template and calculated an eta square value for the network. Each grayordinate is then assigned the network based on the maximum eta^2 value. B) Each grayordinate was assigned a label for each subject. C) The probability of each network was calculated at each grayordinate. D) We thresholded the probability maps to produce a probabilistic ROI set of high network homogeneity using ABCD group1.

ABCD network templates

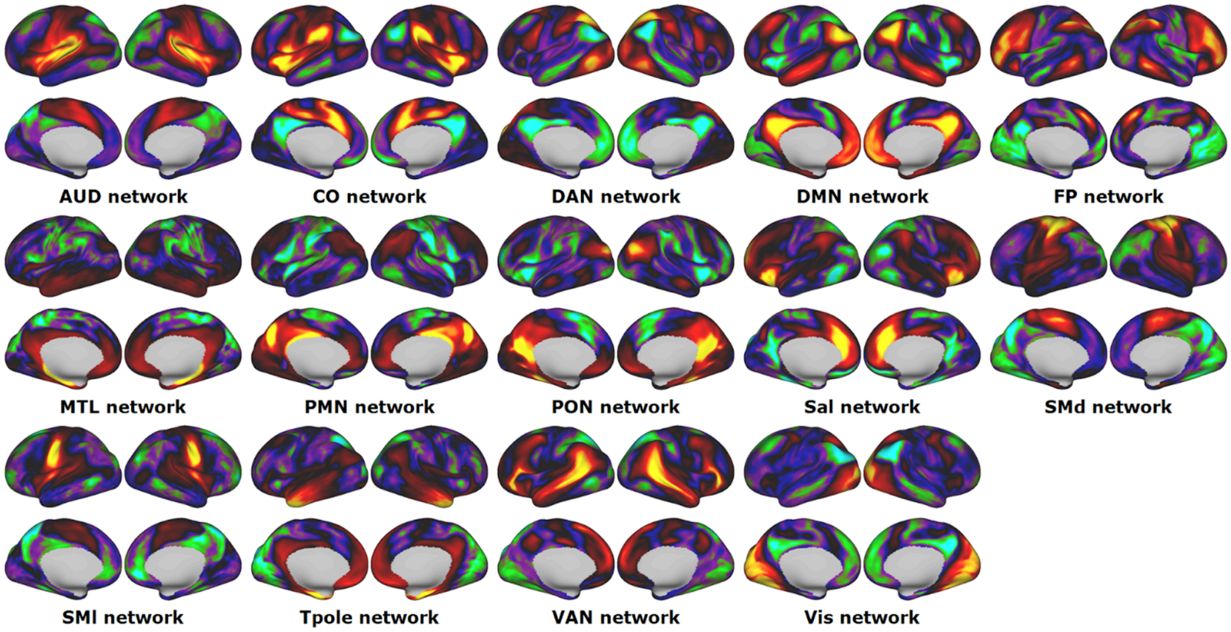

Supplementary Figure S4) Network Templates. Each network that was used to compare functional connectivity to the experimental groups. The list of networks included are the default mode network (DMN, the visual network (VIS), the frontoparietal network (FPN), the dorsal attention network (DAN), the ventral attention network (VAN), the salience network (Sal), the cingulo-opercular network (CO), the dorsal sensorimotor network (SMd), the lateral sensorimotor network (SMl), the auditory network(AUD), the temporal pole network (TP), the medial temporal network (MTL), the parietal occipital network (PON), and the parietal medial network (PMN).

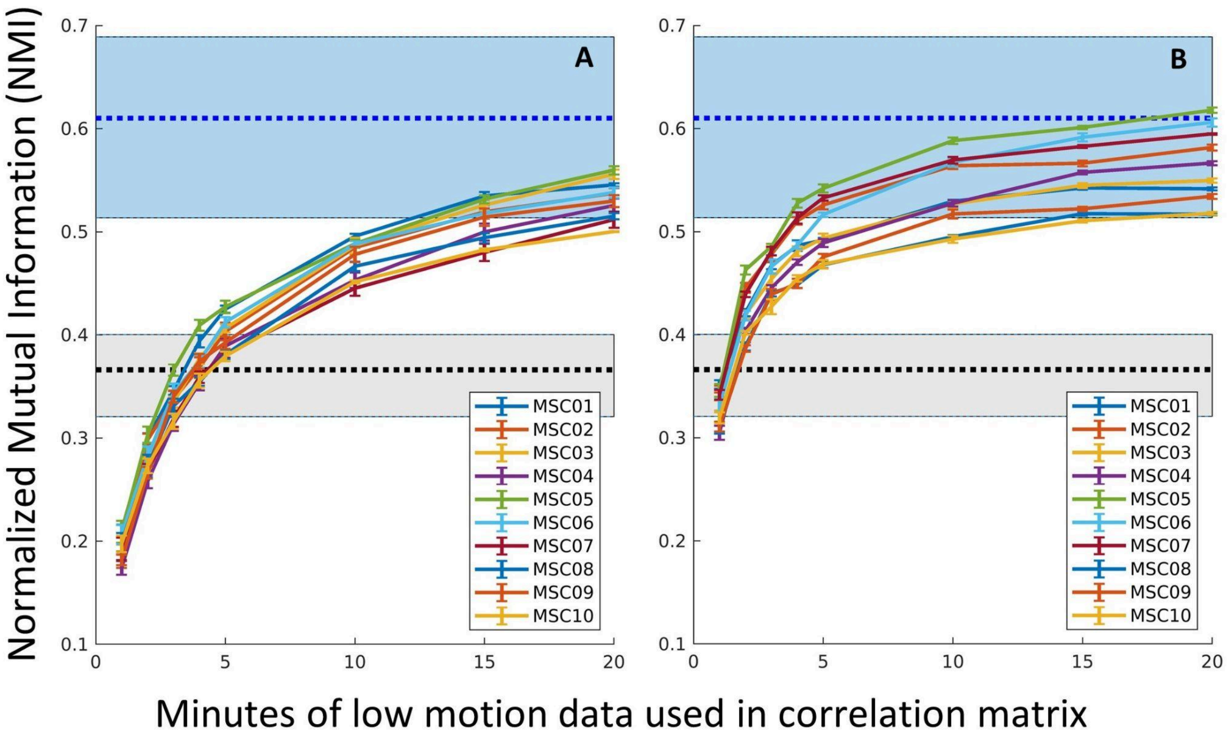

Supplementary Figure S5: Calculating networks generated at various time intervals. Using data from the Midnight Scan Club (MSC) (n=10) who underwent 5 hours of resting state fMRI (in addition to task collection)[1–4]. We calculated the NMI between network maps generated from either A) continuous low-motion time frames or B) randomly-sampled low-motion frames, to the participant's own hold-out half. The range of the maximum NMI for between participant's own halves using all available motion-censored data is shaded in blue, and the NMI to other participants in the group is shaded in gray. The shaded region indicates the range of the distribution of NMI, using the maximum amount of low motion data for each half, and the thick dotted line represents the mean. In A and B, each colored tracing represents 1 participant. Error bars denote ±1 standard error from 10 random samplings from the time interval sampled from the participant's own first half.

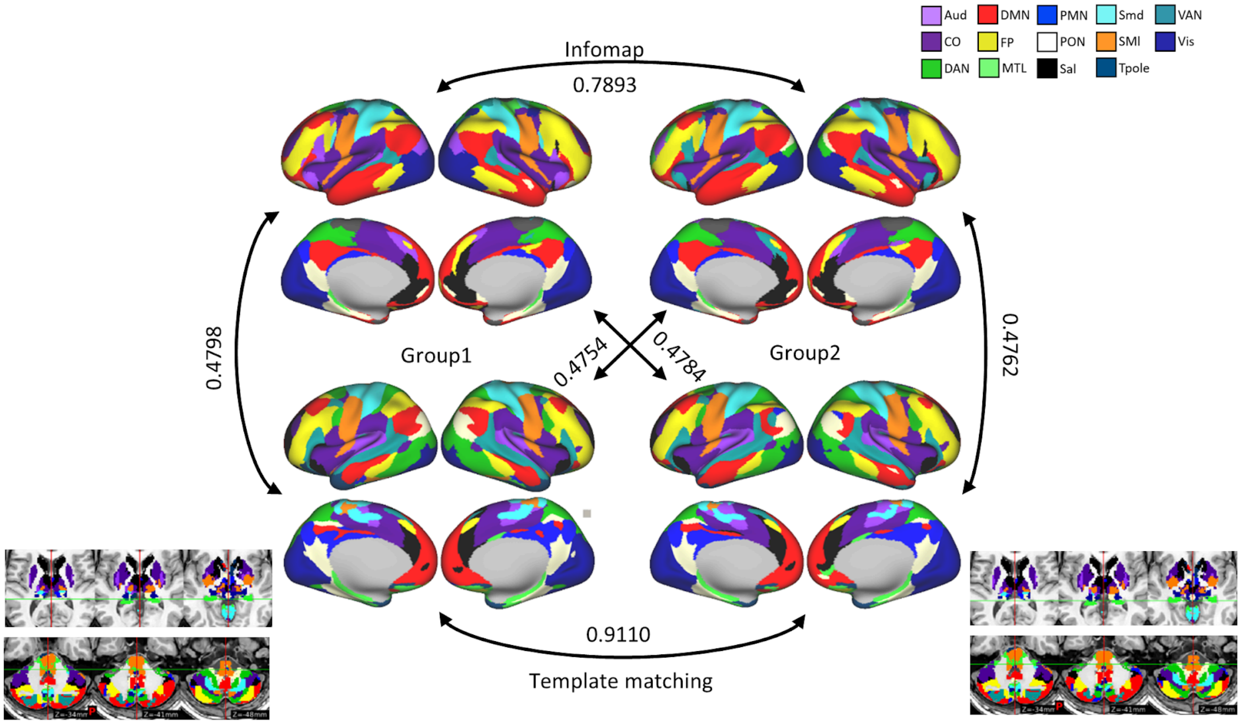

Supplementary Figure S6: Split half group average comparison with resting state data. Infomap and template matching were run on identical connectivity matrices generated from matched groups. We demonstrate the infomap (upper row) and template matching (lower row) produced relatively high replication as evidenced by split group NMI. Insets: networks labels identified for subcortical regions and cerebellum.

Overlapping networks example

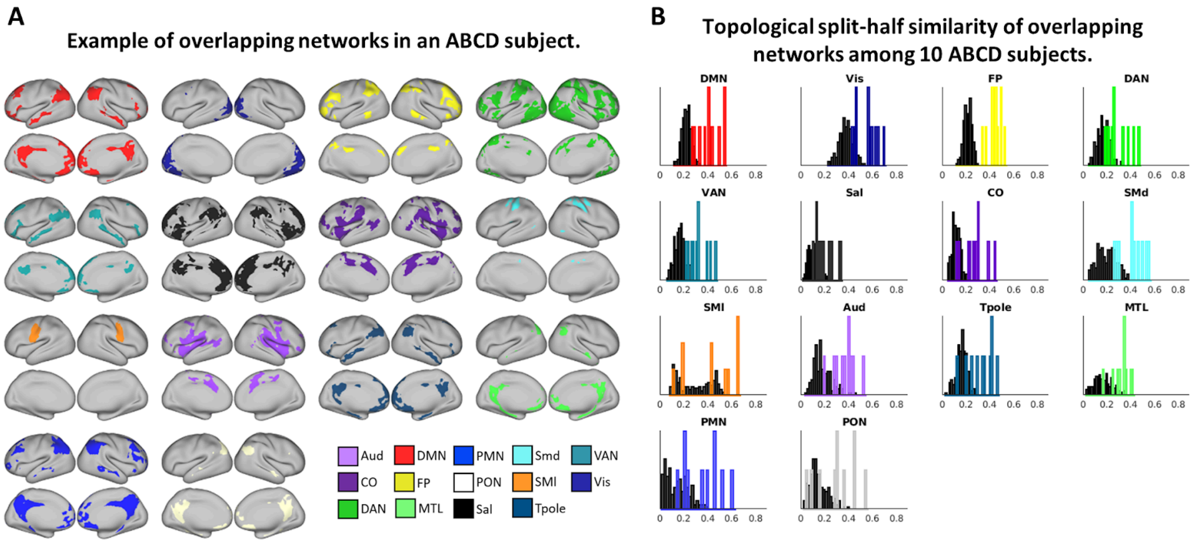

Supplementary Figure S7: Overlapping networks from OMNI Mapping. Networks were identified using

cortical and subcortical regions. Grayordinates with an eta^2 value above the threshold for multiple networks (see Overlapping MultiNetwork Imaging (OMNI) mapping template matching method in *Methods* in main text) received multiple network assignments (see Figure 6 in main text for method). A) Example of overlapping network maps in an ABCD subject. Each network is shown separately for visual comparison. Overlapping networks are shown for a subject with 10 minutes of low motion resting state data. Networks were identified using cortical and subcortical regions. grayordinates with an eta^2 value that is high for multiple networks can therefore receive multiple network assignments (see Figure 6 in main text for method). B) For the 10 subjects 20 minutes of low motion data, we performed a similar NMI analysis to that shown in Figure 3 (main text), but we calculated the NMI for each network separately. Histogram heights have been normalized such that the area under the curve is equal. Note that for networks where topography is highly conserved (such as the lateral somatomotor network), there is considerable overlap of the distributions of NMI of networks for the same vs different subjects. Conversely, the NMI for corresponding halves in networks with highly individualized topology (such as the frontoparietal network) is well outside the null distribution.
Colors for networks are identical to those shown in Figure 1.

Probabilistic maps

Probabilistic maps - single network - Template matching: 10 minutes of resting-state data (surface and subcortical)

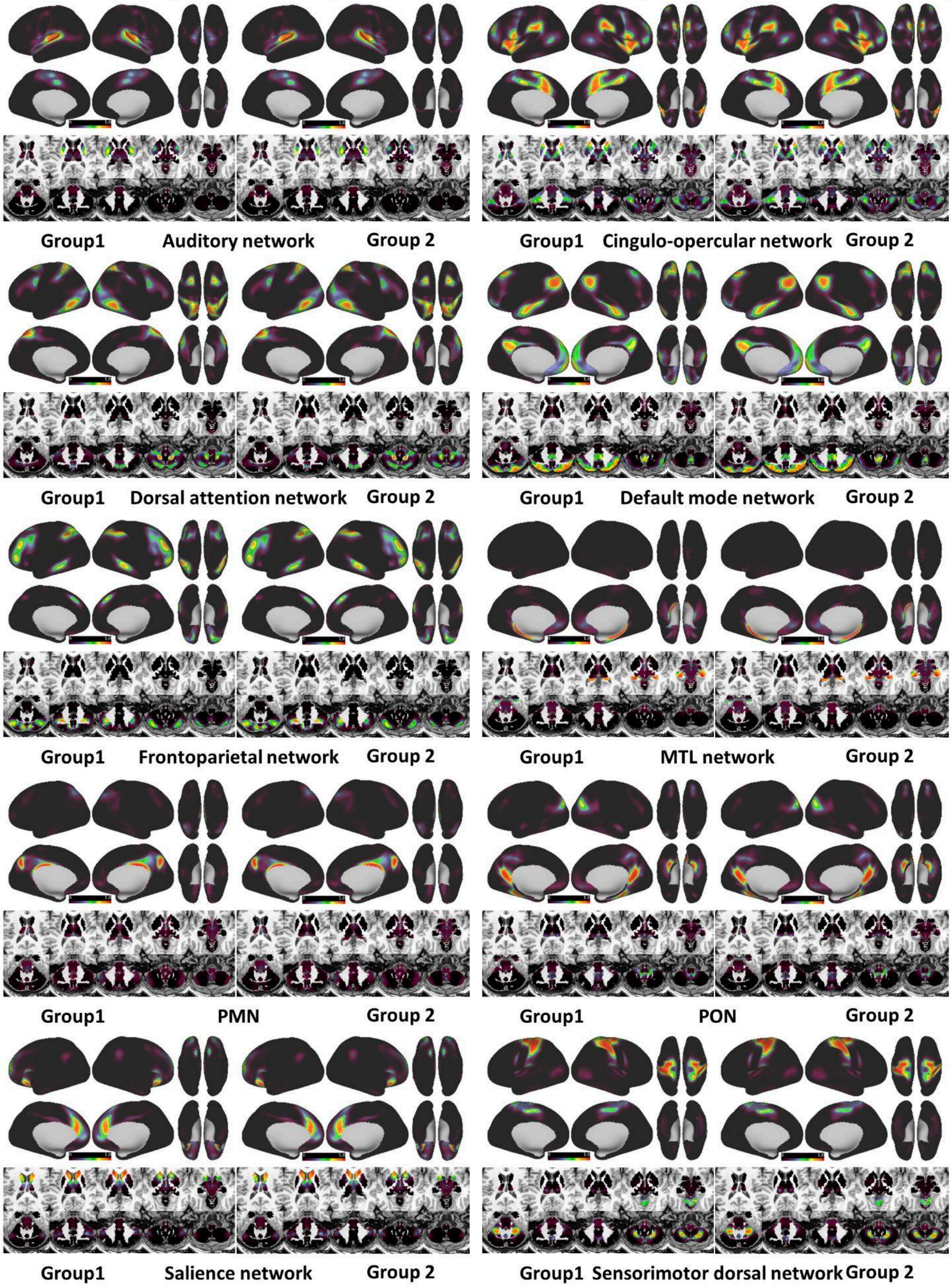

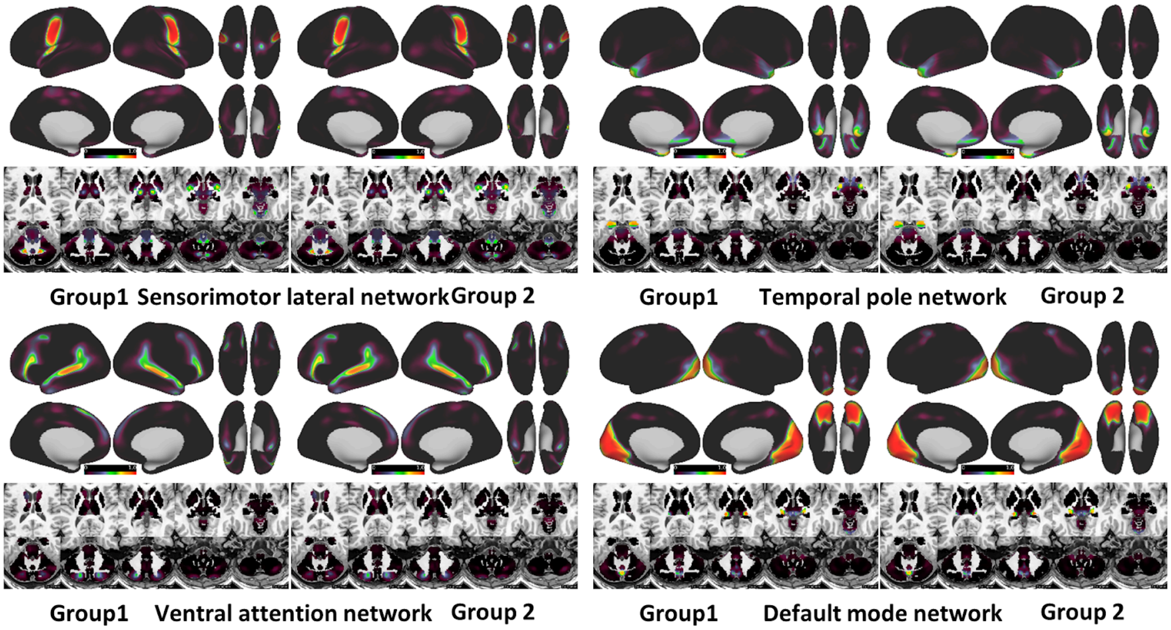

Supplementary Figure S8: Probabilistic rest maps (single network). An example of the probabilistic maps generated by template matching and Infomap algorithms for the frontoparietal cortex is shown in the main text of this manuscript (Figure 2). Here we show the remainder of those maps. Note the strong replication between methods and groups.

Probabilistic maps – single networks - rest using 10 minutes (cortex only)

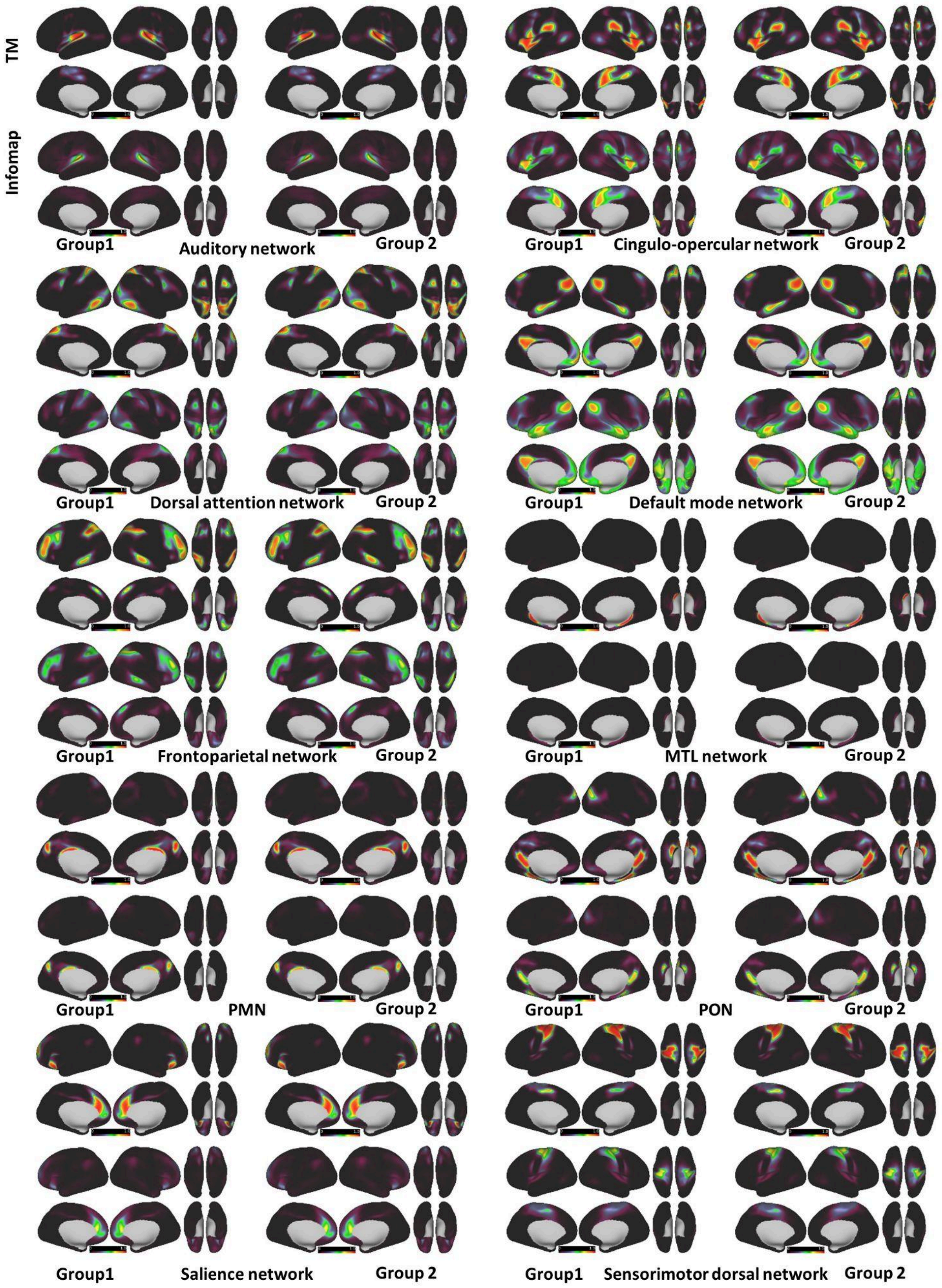

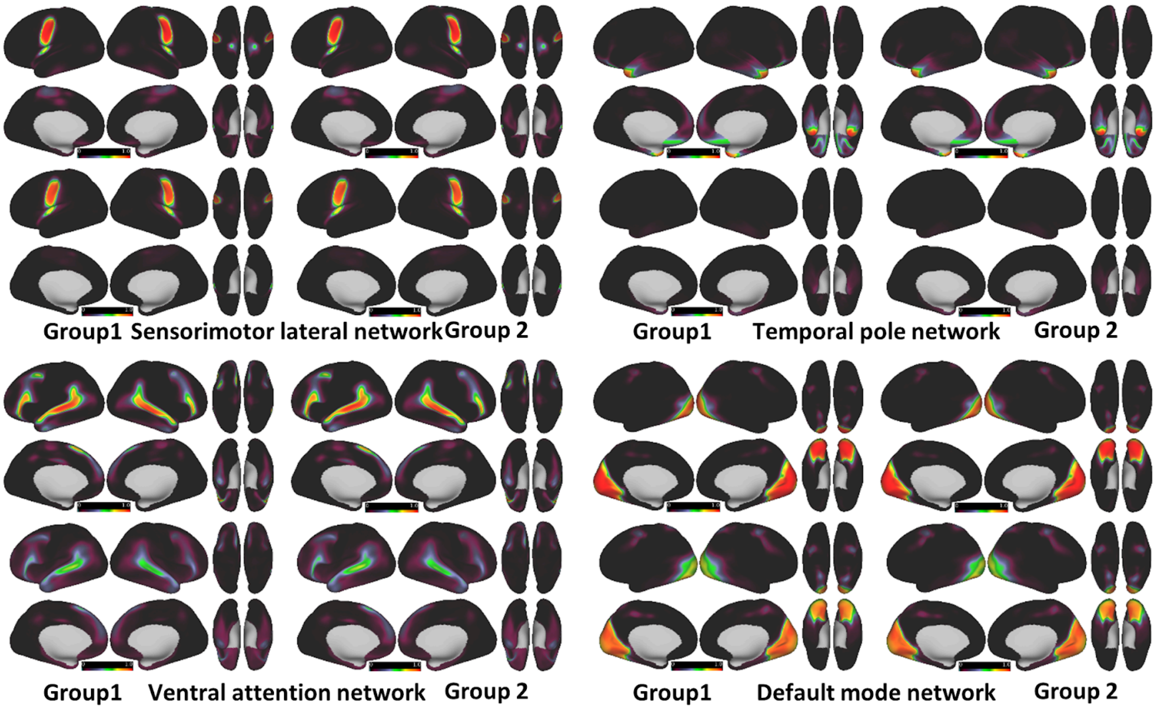

Supplementary Figure S9: Probabilistic task maps (single network). The same probabilistic network maps as those shown in Supplementary Figure S8 except that networks were identified using only data from the cortex. This allowed us to compare the topological similarity of our findings to previously identified networks in the literature which have also constrained analysis to the cortex [3]. The probabilistic maps shown in figure S2 are the same as those shown in figure S1, except that only the correlation data from the cortical surface was used. Infomap community detection was also conducted and is shown here as well (lower images).The range of all maps is identical (0-1).

Supplementary table 1: Correlation between group probabilistic maps using REST data using template matching. *MMZ indicates mismatched zeros. MMZ were excluded from the Pearson correlation shown.

	Template matching				Infomap		Between Methods Single network			
	Single Net		Overlapping		Single Net		Group1		Group2	
	r	%MMZ	r	%MMZ	r	%MMZ	r	%MMZ	r	%MMZ
Aud	0.9997	0.119	0.9994	0.1259	0.9994	0.0044	0.9142	0.404	0.914	0.3794
CO	0.9997	0.0917	0.9993	0.0986	0.9993	0	0.9184	0.228	0.9186	0.2178
DAN	0.9997	0.1016	0.9994	0.0921	0.9991	0.0351	0.9468	0.2471	0.9485	0.2641
DMN	0.9996	0.0935	0.9994	0.1317	0.9994	0	0.7019	0.301	0.7071	0.3042
FP	0.9996	0.0925	0.9994	0.1339	0.9991	0.0045	0.9613	0.2407	0.9633	0.2295

MTL	0.9998	0.2115	0.9999	0.2673	0.9916	0.0452	0.8465	0.4421	0.8511	0.4501
PMN	0.9995	0.1045	0.9997	0.1314	0.9994	0.025	0.9564	0.1664	0.957	0.154
PON	0.9995	0.1147	0.9993	0.1069	0.9991	0.0033	0.9294	0.238	0.9299	0.2398
Sal	0.9996	0.1458	0.9993	0.2136	0.9988	0.0096	0.9093	0.3808	0.9099	0.3665
SMd	0.9998	0.139	0.9997	0.187	0.9994	0.012	0.9564	0.3833	0.9538	0.3356
SMl	0.9996	0.1411	0.9998	0.0941	0.9998	0.1669	0.9461	0.2289	0.9441	0.2812
Tpole	0.9996	0.1456	0.9992	0.1372	0.9961	0.0761	0.6792	0.2924	0.6734	0.2719
VAN	0.9996	0.0973	0.9994	0.1402	0.9987	0.005	0.9446	0.2891	0.943	0.2619
Vis	0.9999	0.1139	0.9996	0.1095	0.9998	0.0968	0.974	0.3566	0.9747	0.394

Supplementary Table 1. We've calculated the similarity between groups and methods using only cortical resting state data (as shown in Supplementary Figure S9). We quantified the Pearson correlation between the probabilistic maps that we observed between Groups 1 and 2, and between methods for each group. For any single network, there were a large number of grayordinates with that had zero probability of being labeled as that network. To reduce bias in the intergroup correlation, we only correlated non-zero elements, but we still quantified the number of mismatched zeros (MMZ) to capture subtle differences in topology.

Probabilistic maps – single networks - Template matching TASK (all low motion data –cortex only)

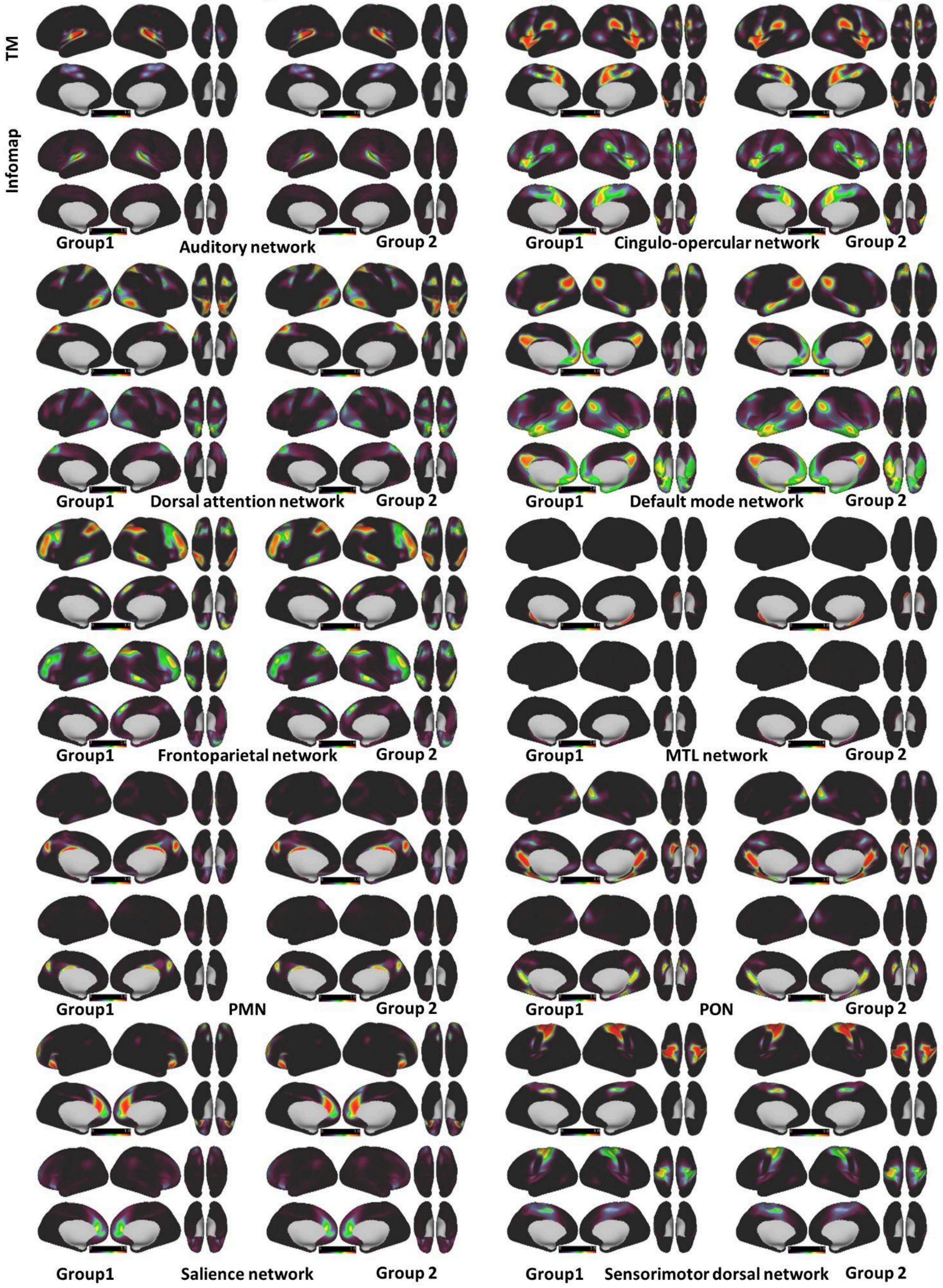

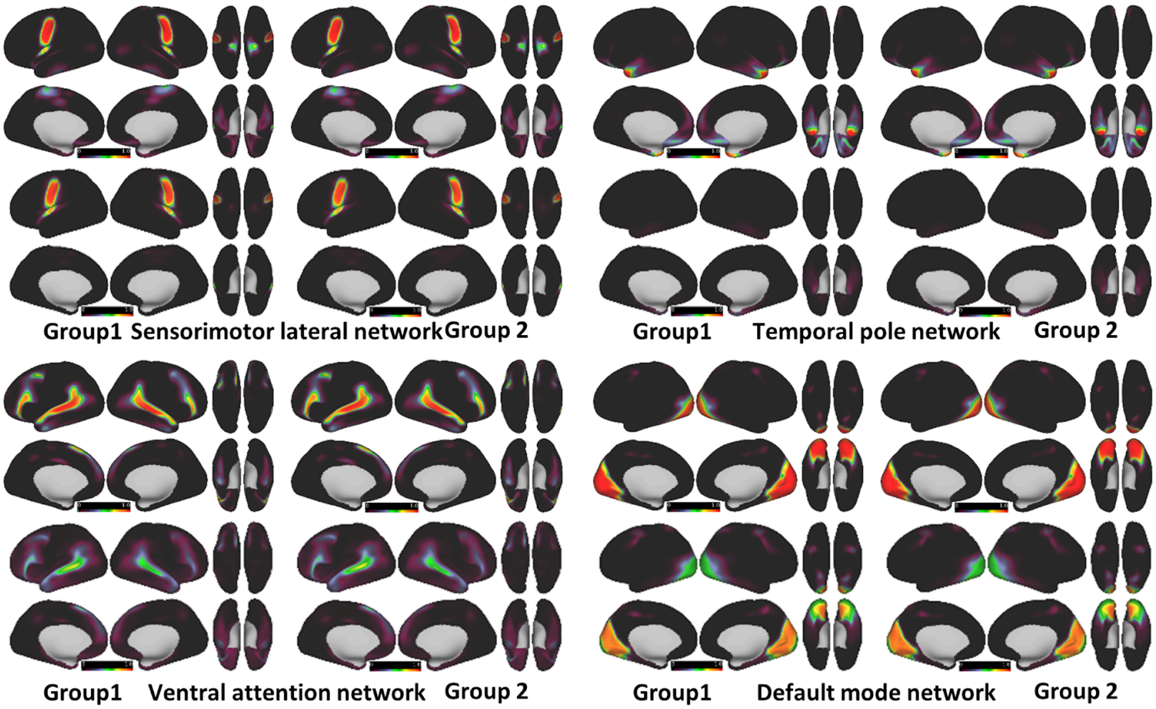

Supplementary Figure S10: Probabilistic task maps (single network assignment) using template matching. The probabilistic maps shown in figure S2 are the same as those shown in figure S1, except that instead of only using resting state data to generate data, concatenated rest and task data was used. The range of all maps is identical (0-1).

Supplementary Table 2: Correlation Between probabilistic maps for each group using TASK data. *MMZ indicates mismatched zeros. MMZ were excluded from the Pearson correlation shown.

	Template matching				Infomap		Between Methods Single network			
	Single Net		OMNI mapping		Single Net		Group1		Group2	
	r	%MMZ	r	%MMZ	r	%MMZ	r	%MMZ	r	%MMZ
Aud	0.9997	0.1224	0.9995	0.1254	0.9993	0.0046	0.8979	0.5251	0.8971	0.4968
CO	0.9996	0.0911	0.9993	0.1332	0.9993	0.0000	0.8939	0.3247	0.8941	0.3142
DAN	0.9996	0.1011	0.9996	0.0988	0.9993	0.0185	0.9371	0.3603	0.9375	0.3553
DMN	0.9995	0.0853	0.9994	0.0785	0.9993	0.0001	0.6858	0.3499	0.6754	0.3612
FP	0.9996	0.0987	0.9994	0.1275	0.9991	0.0025	0.9508	0.3155	0.9538	0.2839
MTL	0.9999	0.2075	0.9999	0.1919	0.9885	0.0476	0.7972	0.6120	0.7913	0.5766
PMN	0.9994	0.1108	0.9996	0.1265	0.9995	0.0097	0.9159	0.2213	0.9172	0.1995
PON	0.9995	0.1023	0.9995	0.1128	0.9994	0.0076	0.9025	0.3181	0.9031	0.3082
Sal	0.9996	0.1413	0.9994	0.2228	0.9986	0.0061	0.8798	0.4694	0.8895	0.4606
SMd	0.9998	0.1419	0.9996	0.1177	0.9995	0.0979	0.9477	0.4556	0.9474	0.4099
SMl	0.9996	0.1251	0.9998	0.0846	0.9998	0.1548	0.8819	0.2406	0.8791	0.2518

Tpole	0.9996	0.1635	0.9993	0.1281	0.9958	0.0901	0.6810	0.3975	0.6752	0.3593
VAN	0.9997	0.0966	0.9993	0.1796	0.9987	0.0056	0.9500	0.3736	0.9523	0.3283
Vis	0.9999	0.1053	0.9996	0.1098	0.9997	0.1096	0.9555	0.4335	0.9557	0.3990

Supplementary Table 2. We've summarized the similarity between groups and methods using the concatenated rest and task data (as shown in Supplementary Figure S9) constrained to the cortex, similar to what is shown in Supplementary Table 1. We quantified the Pearson correlation between the probabilistic maps that we observed between Groups 1 and 2, and between methods for each group. Though inter-method correlations are slightly lower compared to rest, between method correlations were still high (0.8769± 0.91 s.d. group1, 0.8769± 0.95 s.d. group2). Less than 1% of grayordinates had mismatched zero probability between groups for any given network.

Probabilistic maps – Template matching Overlapping Networks: 10 minutes of resting-state data

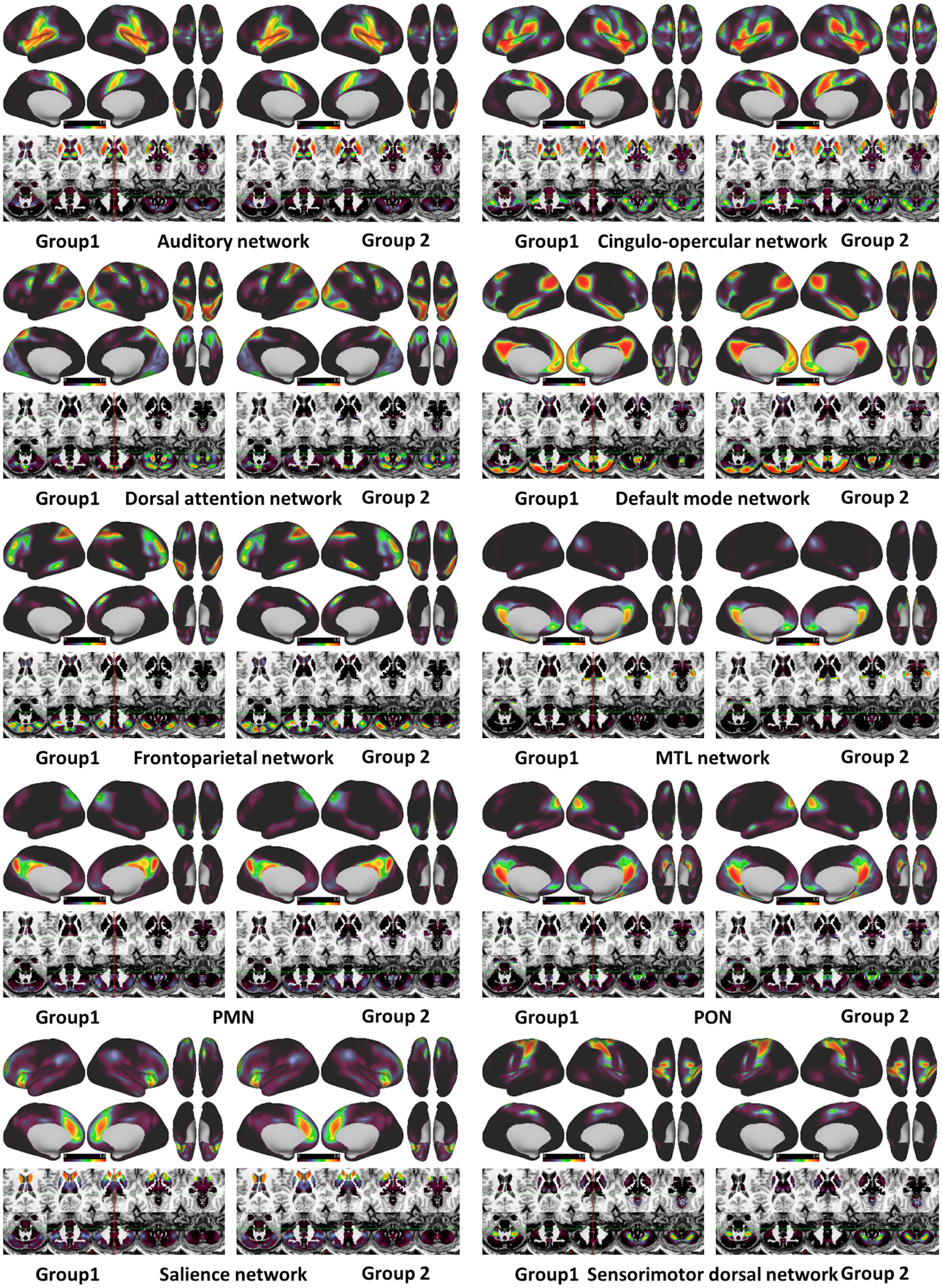

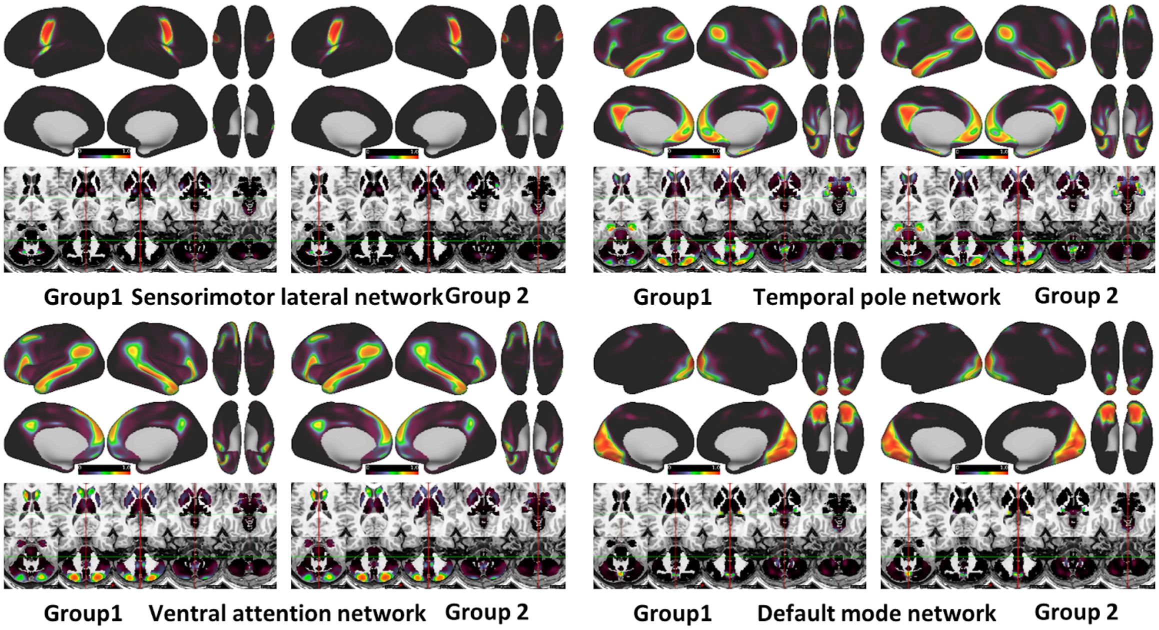

Supplementary Figure S11: Probabilistic Rest maps with overlapping networks using OMNI mapping. We generated probabilistic maps with overlapping networks for resting state data. These maps are similar to the maps shown in Supplementary Figure S8 except that network assignment was allowed to overlap. Note that the region of high probability is generally larger than those shown in Figure S8. Network maps were generated using the procedure outlined in figure 5 using 10 minutes low-motion of resting state data. The range of all maps is identical (0-1).

Supplementary Table 3: Correlation Between probabilistic maps from NMF community detection for each group using concatenated TASK data. *MMZ indicates mismatched zeros. MMZ were excluded from the Pearson correlation shown.

Network	r	%MMZ
1 Default Mode Network	0.99989	0.353245
2 Somatomotor	0.999897	0.004225
3 Fronto-parietal	0.999698	0.015872
4 Somatomotor	0.99984	0.003148
5 Dorsal Attention	0.999806	0.01153
6 Visual	0.9999	0.003619
7 Ventral Attention	0.999587	0.00574
8 Default Mode Network	0.99971	0.009005

9 Ventral Attention	0.999718	0.013432
10 Visual	0.9999	0.002474
11 Somatomotor	0.999824	0.00409
12 Default Mode	0.999711	0.011984
13 Somatoromotor	0.999899	0.003249
14 Dorsal Attention	0.999631	0.008618
15 Fronto-parietal	0.999609	0.001818
16 Auditory	0.999864	0.014054
17 Frontoparietal	0.999741	0.011227

Supplementary Table 3: To compare the similarity of the probabilistic maps between each ABCD group, we correlated probabilistic maps from NMF community detection for each network using concatenated REST+TASK data.

Supplementary Table 4: Correlation Between probabilistic maps generated from resting state data only and concatenated task+rest resting state data using template matching for each group. *MMZ indicates mismatched zeros. MMZ were excluded from the Pearson correlation shown.

	Group1		Group2	
	r	%MMZ	r	%MMZ
Aud	0.996851	0.140729	0.996851	0.135646
CO	0.996441	0.107537	0.996526	0.105433
DAN	0.995095	0.130395	0.995265	0.106443
DMN	0.993439	0.076416	0.993926	0.072982
FP	0.992723	0.098246	0.993034	0.075271
MTL	0.990461	0.220191	0.990196	0.18126
PMN	0.983812	0.09621	0.984586	0.092372
PON	0.988855	0.104036	0.988894	0.097068
Sal	0.99597	0.126759	0.996174	0.127281
SMd	0.995415	0.12346	0.995017	0.127129

SMl	0.982372	0.119673	0.982476	0.108547
Tpole	0.991341	0.16633	0.992338	0.131926
VAN	0.995826	0.092288	0.99588	0.085286
Vis	0.996753	0.104558	0.996811	0.097842

Supplementary Table 4. In addition to correlating probabilistic maps across groups and methods, we also correlated probabilistic maps generated with resting state data alone and concatenated rest+task. Note that more subjects were used in the task+rest dataset compared to rest alone. The very high correlation between the probabilistic maps reinforces recent evidence that global network topography is mostly unchanged even under task conditions. The strong correspondence between network maps between task and rest+task is supported by recent evidence that indicates that single tasks, or even combinations of tasks produce similar network maps to those observed at rest [5].

Volumetric averaging and integration zones

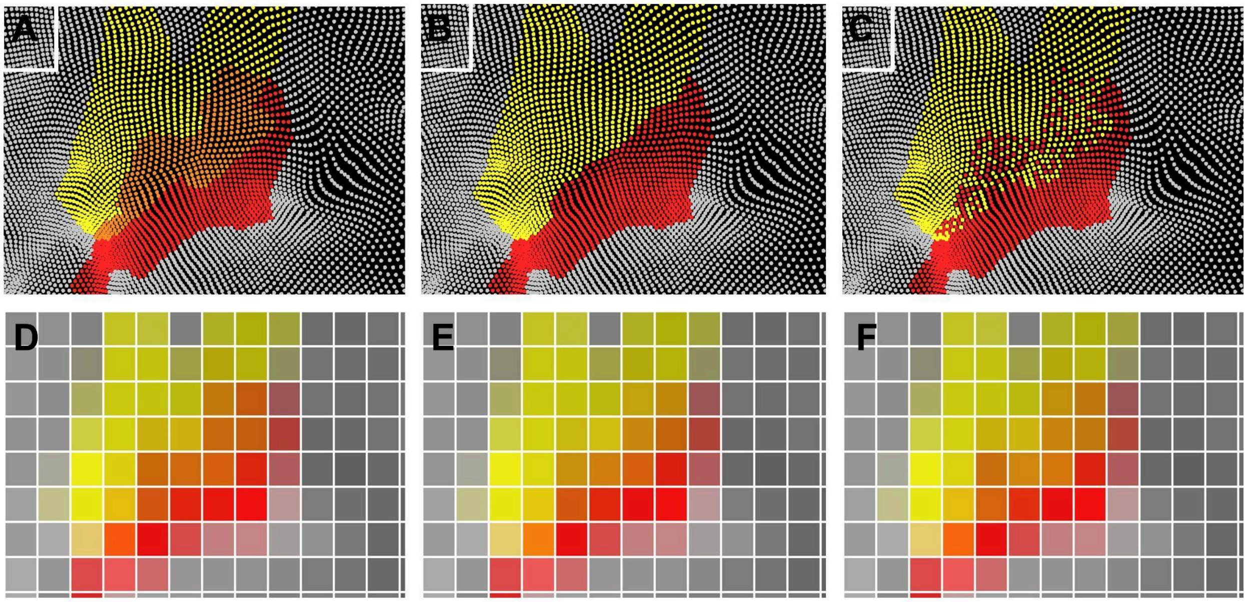

Supplementary Figure S12: Schema for how Integration may arise from different topographical arrangements. White dots represent a functional unit (e.g. a neuron) **A-C)** Examples of various topographical border arrangements. Red and yellow represent separate networks. Orange represents functional units that have shared properties of the red and yellow networks. In A, the functional units in orange participate in both the red and yellow networks. In B, functional units establish discrete borders between networks. In C, functional units participate in distinct networks, but are spatially interdigitated. **D-F)** Volumetric averaging may obscure our ability to distinguish these arrangements (grid not shown to scale for explanatory purposes). Regardless of the specific cytoarchitectural arrangements, functional units that reside at all internetwork boundaries are likely important for integration as well.

Supplementary Table 5. Coefficients for 3-parameter Rise-to-Maximum Fit Coefficient for parcellations shown in Figure 4.					
	Coefficient	MIDB Probabilistic Parcellation (80 ROIs)	Gordon Parcellation Full (352 ROIs)	Gordon Parcellation subset (80 ROIs)	Integration Zone Parcellation (30 ROIs)
PC1	y0	0.1819±0.0078	0.1425±0.0059	0.1471±0.0074	0.1498±0.0079
	a	0.5744±0.0088	0.5843±0.0071	0.5867±0.0090	0.6058±0.0100
	b	0.0031±0.0001	0.0033±0.0001	0.0032±0.0002	0.0031±0.0002
PC2	y0	0.0843±0.0078	0.0639±0.0055	0.0581±0.0074	0.1115±0.0139
	a	0.4959±0.0149	0.4409±0.0110	0.4378±0.0132	0.5987±0.0225
	b	0.0022±0.0002	0.0021±0.0001	0.0022±0.0002	0.0024±0.0003
PC3	y0	0.1239±0.0080	0.1034±0.0053	0.0979±0.0066	0.1254±0.0131
	a	0.5266±0.0109	0.4881±0.0077	0.4812±0.0092	0.5586±0.0193
	b	0.0029±0.0002	0.0027±0.0001	0.0028±0.0002	0.0026±0.0003

Supplementary Table 5 shows the regression coefficient for the reliability analysis (Figure 4) using the different ROI sets.

Motion effects on within-subject reliability

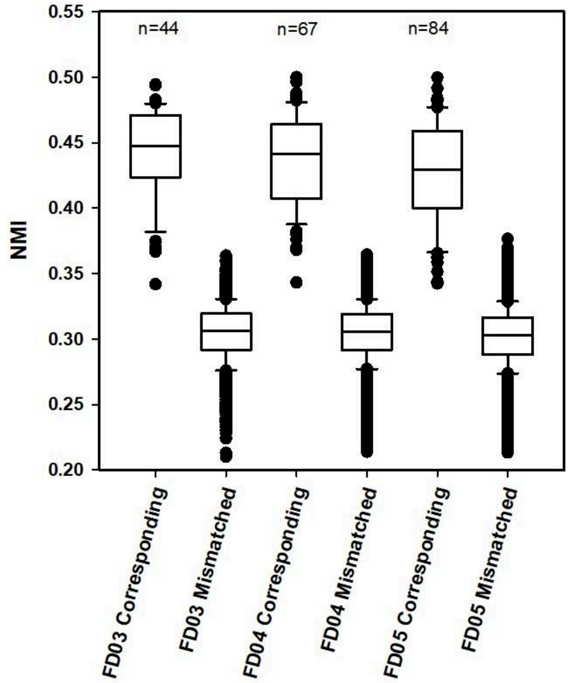

Supplementary Figure S13: Inclusion of additional movement. We explored the extent to which motion affects the individual-specific networks. We generated network maps from split halves (using additional the subjects that had at least 10 minutes of low motion resting state data at an FD<0.3 (real=0.4426+-0.0361, null=0.3044+-0.0218) , FD<0.4 (real=0.4368+-0.0356, null=0.3045+-0.0210), and FD<0.5 (real=0.4275+-0.0392, null=0.3018+-0.0215) . The data suggest that the inclusion of additional motion moves the distribution of the corresponding halves toward the null distribution. However, even with a framewise displacement threshold of FD0.5, in the corresponding halves, only 13% have NMI values that are lower than the maximum value of the mismatched null distribution. NMI=Normalized mutual information. Boxplots show median, interquartile range, IQR (box size). The whisker maximum and minimum whiskers represent Q3+1.5*IQR and Q1-1.5*IQR.

Qualitative assessment spatial performance of template matching

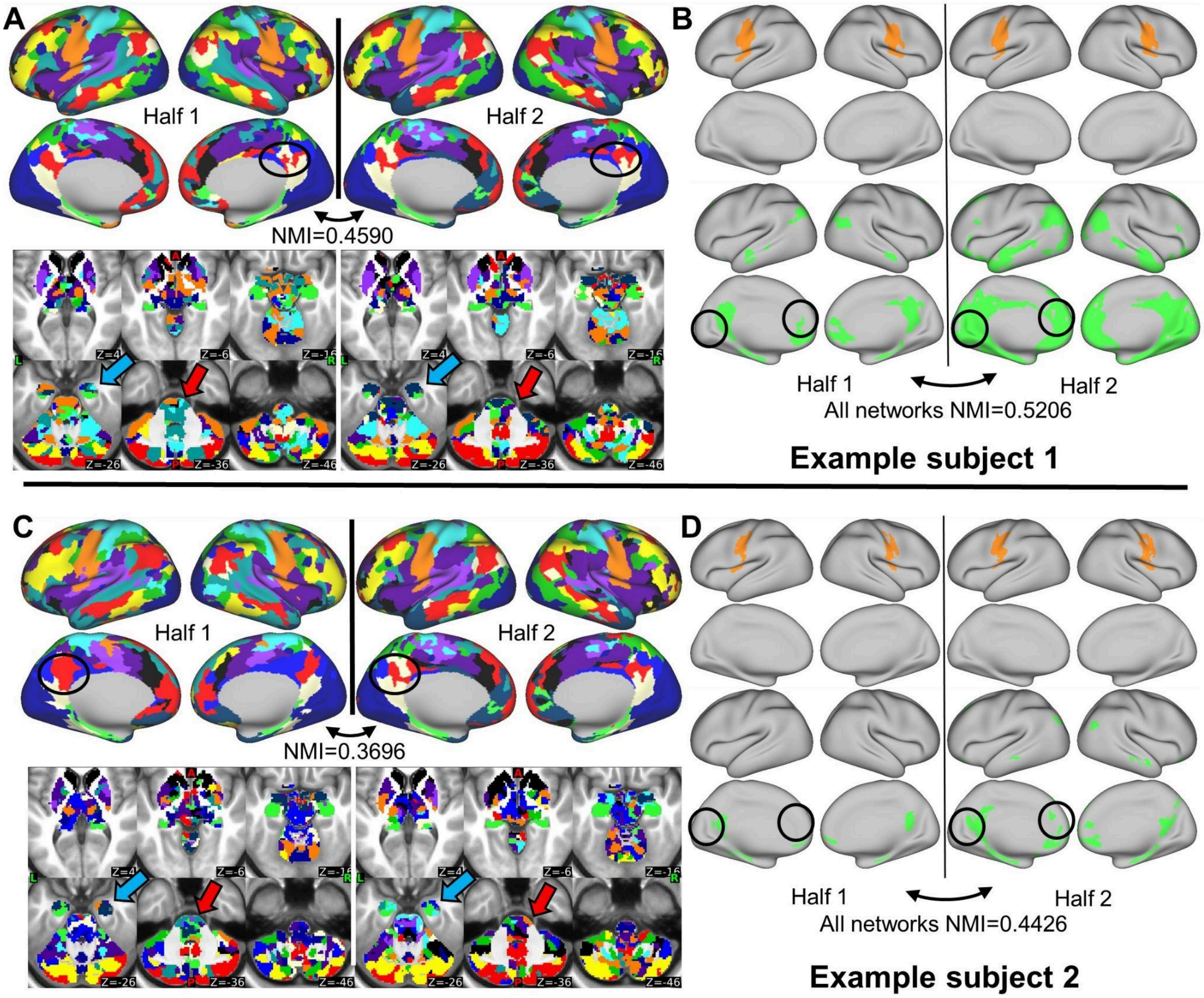

Supplementary figure S14: Examples of within-subject template matching performance using split half analysis. To assess regions where the template matching procedure performs best, we've provided example networks for 2 participants using a FD threshold of 0.2 at 10 minute split halves. Left (A,C): Single network assignments using the template matching method. Right (B,D): Overlapping network assignments using the OMNI mapping. For the single network assignment template matching procedure, template matching produced discordant assignments in regions that either had a high signal-to-noise (e.g. inferior temporal lobe, orbitofrontal cortex, medulla, and pons (A,C, red arrows)) or were heavily connected to multiple regions, such as the posterior cingulate cortex, (A,C black circles) and the hippocampus (A,C, blue arrows). For the OMNI mapping procedure, the procedure performed best with unimodal processing regions such as the lateral somatomotor network (B,D orange networks), and we observed less consistent patterns of connectivity for regions that have either a noisy or nested structure such as the medial temporal network (B,D green networks). In A and C, note the challenges of assigning a single network to the posterior cingulate cortex which communicates with multiple networks, namely default mode network (DMN - red), the parietal occipital network (PON - white), and the parietal medial network (PMN - blue).

Average ABCD parcellated connectivity matrix using HCP-D ROIs

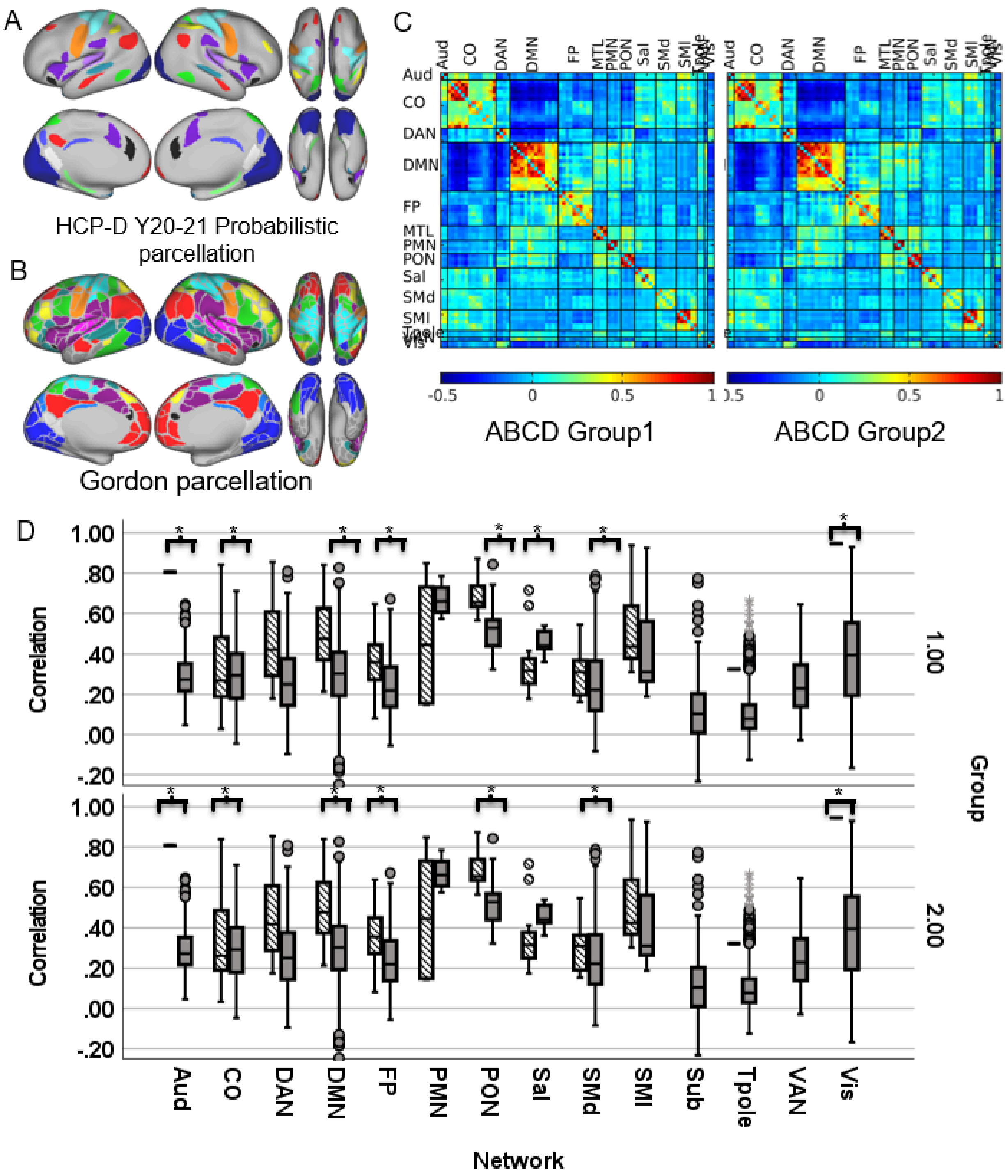

Supplementary Figure S15: Comparing connectivity using HCP-D probabilistic ROIs and Gordon ROIs. In addition to generating probabilistic maps for participants from the ABCD study (ages 9-10), we also generated probabilistic maps from the Human Connectome Project: Developmental (HCP-D). Probabilistic maps from each age group are shown in Figure 6A. **A** To test whether the within-network connectivity findings reported in Figure 3G in the Main text were due to the fact that the probabilistic parcellation was derived from adults and the MIDB probabilistic parcellation (n=80 parcels) was derived from children, we generated parcellated connectivity matrices using a similar parcellation the 20-21 year old group of participants (n=67 participants) from the HCP-D study (n=79 parcels, 75% probability of network consensus). We observed a nearly identical pattern of results to those observed in the analogous analysis in the main text. The correlation matrices were built from using group averaged data from ABCD resting state data for groups 1(n=2995) and 2(n=3111) . **Bottom:** 14 shared networks between the parcellations, showed significantly higher within network connectivity in using the MiDB probabilistic parcellation. Open boxes denote the within network connectivity for the Gordon Parcellation, whereas striped boxes indicate the HCP-D Probabilistic parcellation. T-tests were conducted between methods using within-network connections using group average connectivity matrices (Aud: df=275;CO: df=869;DAN: df=500; DMN: df=909; FP: df=319; PMN: df=14; PON: df=32; Sal: df=19; SMd: df=716; Sml: df=41; Tpole/unlabeled: df=1079; Vis:df=740. The number of ROIs and therefore the number of degrees of freedom are identical for groups 1 and 2.) * indicates alpha < 0.05. Boxplots show median, interquartile range, IQR (box size). The whisker maximum and minimum whiskers represent Q3+1.5*IQR and Q1-1.5*IQR.

Comparison of Sites

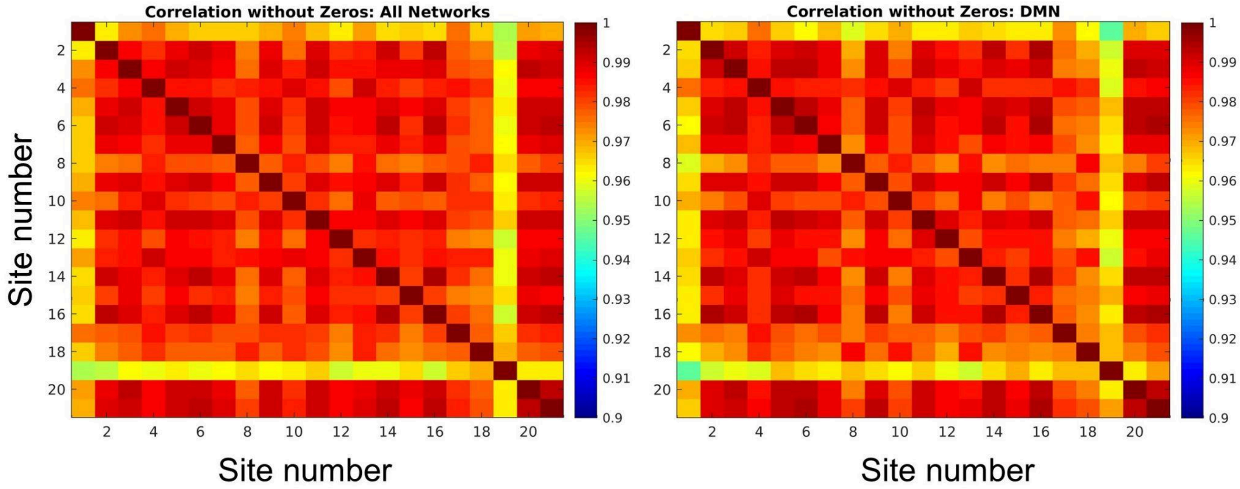

Supplementary Figure S16: Correlation between probabilistic maps between sites. The ABCD study recruited participants from 21 different sites, and therefore probabilistic maps have the potential to be biased by sites that have larger numbers of participants. We therefore generated probabilistic maps from each site independently and correlated the probabilistic maps between each site. We found that probabilistic maps were nearly identical between sites. (Mean correlation between sites for each network: Aud = 0.982±0.011, CO=0.978±0.010, DAN=0.981±0.0096, DMN=0.981±0.010, FP=0.984±0.007, MTL=0.9810.0155±, PMN=0.976±0.011, PON=0.980±0.009, Sal=0.982±0.009, SMd=0.984±0.008, SMl=0.980±0.011, Tpole=0.973±0.017, VAN=0.980±0.009, Vis=0.991 ±0.005). **Left :** This plot shows the correlation (averaged across networks) between each site. **Right:** Example of the between site

correlation of the probabilistic maps for the default mode network. Correlations were calculated excluding “0s” so as to reduce bias in probabilistic maps that have smaller representations.

Split half reliability of probabilistic from broad age ranges.

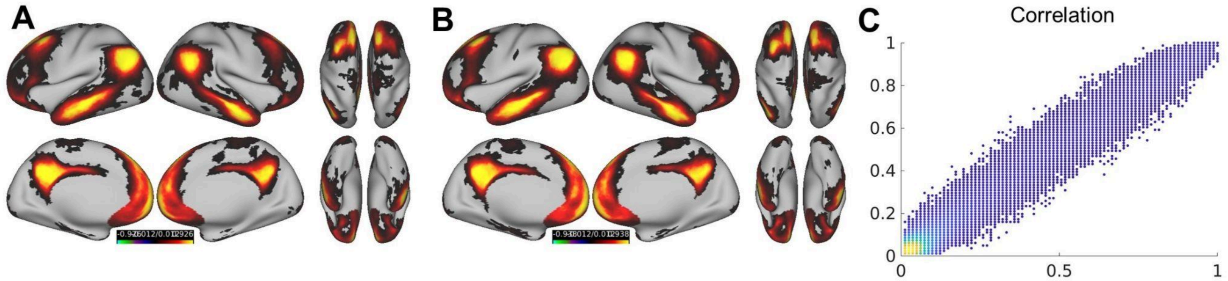

Supplementary Figure S17: Correlation between probabilistic maps for broader age ranges. We divided participants from HCP-D ages 8-13 into 2 cohorts, and generated probabilistic maps for each group. The Default mode for each HCP-D group is shown as an example. The correlation between probabilistic maps for each network is shown below in Supplementary table 7. ***A)*** *Probabilistic maps were generated from half of the subjects within the age range 8-13.* ***B)*** *Probabilistic maps were generated for the remaining subjects. For brevity, only the probabilistic map of the default mode network is shown.* ***C)*** *Correlation between the probabilistic maps shown in A. and B. Correlation was conducted only on grayordinates where both values are not zero. Each dot represents a grayordinate. Colors represent the probability density estimate.*

Supplementary Table 6: Correlation of probabilistic maps using template matching between each HCP-D 08-13 group using concatenated resting data. *MMZ indicates mismatched zeros. MMZ were excluded from the Pearson correlation shown.		
Network	r	%MMZ
Aud	0.9841	0.1531
CO	0.9615	0.1732
DAN	0.9826	.1448
DMN	0.9865	0.1406
FP	0.9801	0.1447
MTL	0.9710	0.1397
PMN	0.9742	0.1721

PON	0.9929	0.1112
Sal	0.9753	0.1165
SMd	0.9766	0.1390
SMl	0.9789	0.1389
Tpole	0.9770	0.1435
VAN	0.9734	0.1387
Vis	0.9778	0.1359

Supplementary Table 7: Correlation between resting state probabilistic maps from template matching HCP-D 08-13 age group and the ABCD 09-10 year old group. *MMZ indicates mismatched zeros. MMZ were excluded from the Pearson correlation shown.

Network	r	%MMZ
Aud	0.9415	0.3105
CO	0.9674	0.2330
DAN	0.9651	0.3198
DMN	0.9421	0.2307
FP	0.9654	0.3831
MTL	0.7292	0.3013
PMN	0.9567	0.4624
PON	0.9540	0.4197
Sal	0.9508	0.3367
SMd	0.96508	0.3050
SMl	0.9645	0.2926
Tpole	0.8488	0.4086
VAN	0.9570	0.3540
Vis	0.9801	0.3007

Comparison to Yeo Confidence map.

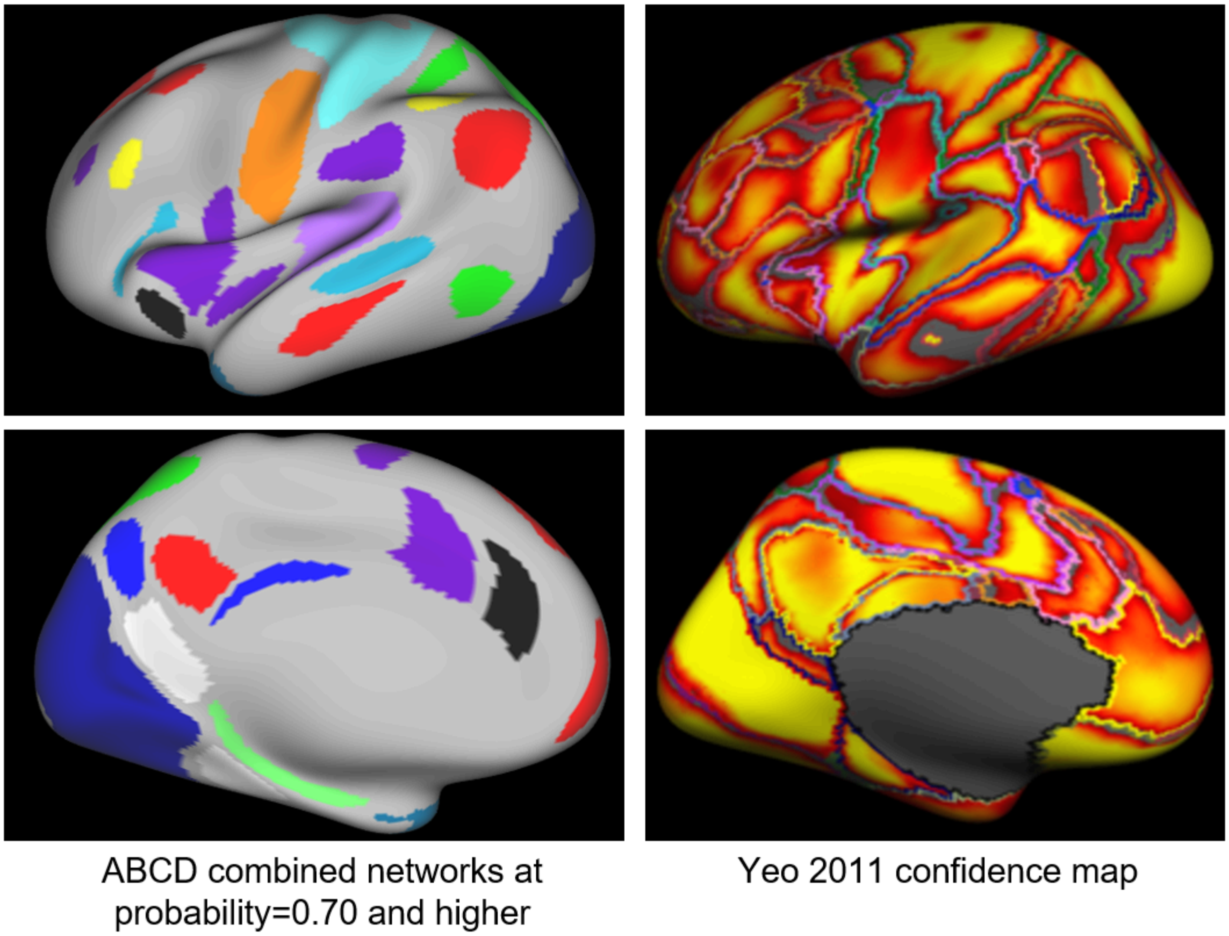

Supplementary Figure S18: Comparison of ABCD probabilistic maps with Yeo parcellation. Others have explored various methodologies to characterize robustness of parcellations. Yeo and colleagues [6] used a silhouette measure that characterizes the similarity of adjacent data points using both a 7 network and 17 network solution. For a comparison to 17 network solution comparison we used the probability of the observed network at a threshold of probability=0.7 and above template matching networks (n=14 networks). There are several differences between these maps, notably the default mode network in the frontoparietal region is markedly dissimilar. Furthermore, the lateral somatomotor network, which we observed to be very consistent across the population, was more markedly dissimilar in the Yeo confidence map which has merged with the auditory cortex. **Left :** ROI set where 70% of the ABCD group1 retained the mode of each grayordinate. **Right:** Confidence map from Yeo and colleagues [6]. Each grayordinate indicates a silhouette measure indicating the similarity of the data to its neighbor.

Topographic reliability in adolescent samples.

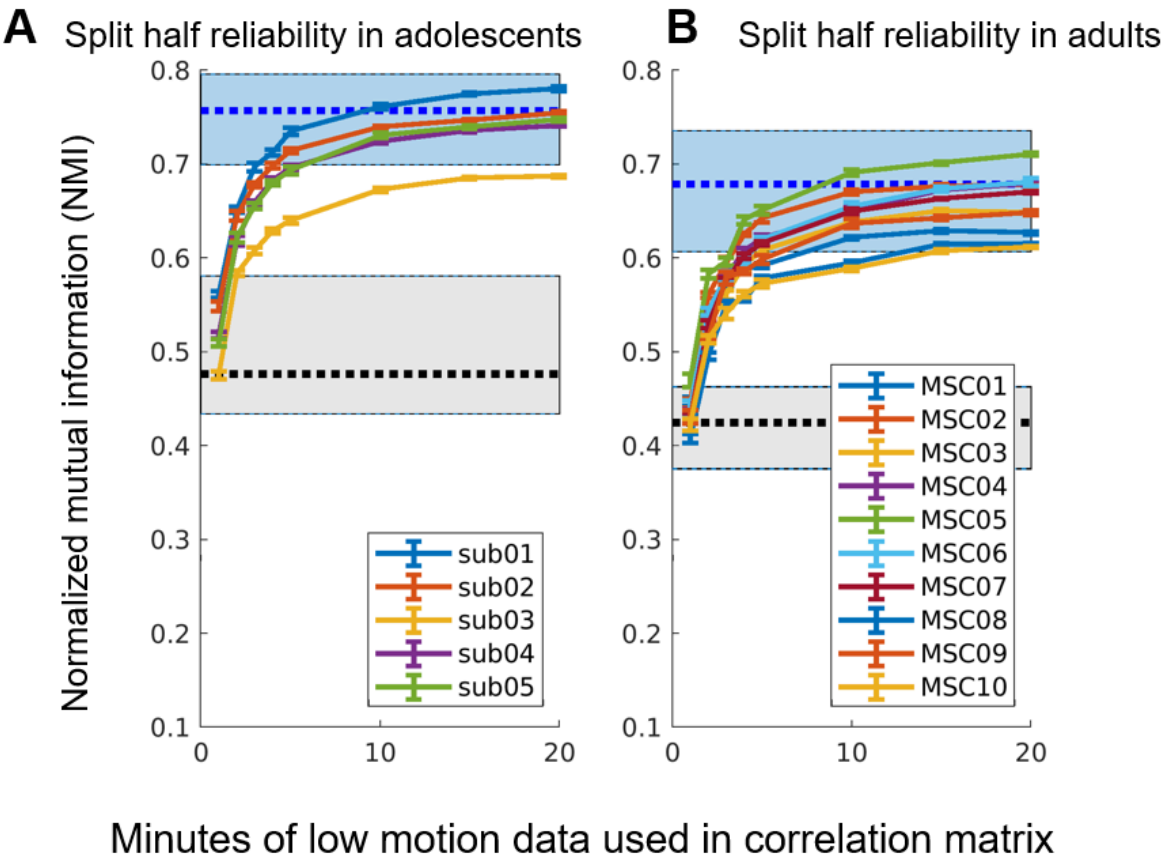

Supplementary Figure S19: Calculating networks generated at various time intervals.
Using data from the MIDB subpopulation cohort (n=5) (A) and Midnight Scan Club (MSC) (n=10) (B) who underwent 2.5 and 5 hours of resting state fMRI respectively [1–4]. We calculated the NMI between network maps generated from randomly-sampled low-motion frames, to the participant's own hold-out half. The range of the maximum NMI for between participant's own halves using all available motion-censored data is shaded in blue, and the NMI to other participants in the group is shaded in gray. The shaded region indicates the range of the distribution of NMI, using the maximum amount of low motion data for each half, and the thick dotted line represents the mean. Each colored tracing represents 1 subject. Error bars denote ±1 standard error from 10 random samplings from the time interval sampled from the participant's own first half.

SUPPLEMENTAL REFERENCES

1. Sylvester, C. M. *et al.* Individual-specific functional connectivity of the amygdala: A substrate for precision psychiatry. *Proc. Natl. Acad. Sci. U. S. A.* **117**, 3808–3818 (2020).

2. Greene, D. J. *et al.* Integrative and Network-Specific Connectivity of the Basal Ganglia and Thalamus

Defined in Individuals. *Neuron* **105**, 742–758.e6 (2020).

3. Gordon, E. M. *et al.* Precision Functional Mapping of Individual Human Brains. *Neuron* **95**, 791–807.e7 (2017).

4. Gratton, C. *et al.* Functional Brain Networks Are Dominated by Stable Group and Individual Factors, Not Cognitive or Daily Variation. *Neuron* **98**, 439–452.e5 (2018).

5. Kraus, B. T. *et al.* Network variants are similar between task and rest states. *Neuroimage* **229**, 117743 (2021).

6. Yeo, B. T. T. *et al.* The organization of the human cerebral cortex estimated by intrinsic functional connectivity. *J. Neurophysiol.* **106**, 1125–1165 (2011).
