# Supplementary material for: A precision functional atlas of personalized network topography and probabilities
Source: Nat Neurosci. 2024 Mar 26;27(5):1000–13. doi: 10.1038/s41593-024-01596-5 (PMC11089006; doi:10.1038/s41593-024-01596-5)
Supplement: Supplementary file 2 — Reporting Summary [file 41593_2024_1596_MOESM2_ESM.pdf]

Corresponding author(s): Robert Hermosillo

Last updated by author(s): 12/11/2023

Reporting Summary

Nature Research wishes to improve the reproducibility of the work that we publish. This form provides structure for consistency and transparency in reporting. For further information on Nature Research policies, see Authors & Referees and the Editorial Policy Checklist.

Statistics

For all statistical analyses, confirm that the following items are present in the figure legend, table legend, main text, or Methods section.

n/a	Confirmed	
☐	☒	The exact sample size (*n*) for each experimental group/condition, given as a discrete number and unit of measurement
☐	☒	A statement on whether measurements were taken from distinct samples or whether the same sample was measured repeatedly
☐	☒	The statistical test(s) used AND whether they are one- or two-sided<br>*Only common tests should be described solely by name; describe more complex techniques in the Methods section.*
☐	☒	A description of all covariates tested
☐	☒	A description of any assumptions or corrections, such as tests of normality and adjustment for multiple comparisons
☐	☒	A full description of the statistical parameters including central tendency (e.g. means) or other basic estimates (e.g. regression coefficient) AND variation (e.g. standard deviation) or associated estimates of uncertainty (e.g. confidence intervals)
☐	☒	For null hypothesis testing, the test statistic (e.g. *F*, *t*, *r*) with confidence intervals, effect sizes, degrees of freedom and *P* value noted<br>*Give P values as exact values whenever suitable.*
☒	☐	For Bayesian analysis, information on the choice of priors and Markov chain Monte Carlo settings
☒	☐	For hierarchical and complex designs, identification of the appropriate level for tests and full reporting of outcomes
☐	☒	Estimates of effect sizes (e.g. Cohen's *d*, Pearson's *r*), indicating how they were calculated

Our web collection on statistics for biologists contains articles on many of the points above.

Software and code

Policy information about availability of computer code

Data collection	FRIMM software (v3.0.0-3.2.13) for real-time motion monitoring (Nous imaging) was used during data collection to provide feedback to the MRI operators about participant motion during data collection.

Data analysis	DCAN-Labs/abcd-hcp-pipeline: https://github.com/DCAN-Labs/abcd-hcp-pipeline - End-to-end pipeline used to perform surface-based analysis.<br>Custom template matching code: https://github.com/DCAN-Labs/compare_matrices_to_assign_networks - used to identify individual specific networks.<br>Connectivity matrix code: https://github.com/DCAN-Labs/cifti-connectivity - This code is to generate correlation matrices.<br>MATLAB v2016b-2021a (Mathworks) https://www.mathworks.com/ - Primary software to write custom code.<br>Connectome Workbench: (Marcus et al., 2011) http://www.humanconnectome.org/software/connectome-workbench - Software to use ciftis for surface-based analysis.<br>Freesurfer (Fischl, 2012): https://surfer.nmr.mgh.harvard.edu/ - Used to generate surface mesh objects for BOLD registration.<br>Infomap Rosvall and Bergstrom, 2008 (Rosvall & Bergstrom, 2008): www.mapequation.org - Community detection algorithm for network validation.<br>FSL 5.0 or greater: (Smith et al., 2004) https://fsl.fmrib.ox.ac.uk/fsl/fslwiki - Neuroimaging tools for working with NIFTIs.<br>Midnight Scan club (Gordon, Laumann, Gilmore, et al., 2017) https://www.openfmri.org/dataset/ds000224/ - Open source adult dataset to validate within-subject reliability.<br>ABCC Collection for matched groups Feczko et al. 2021. https://www.biorxiv.org/content/10.1101/2021.07.09.451638v1 - Used to generate matched groups.<br>fMRIPrep (v23.0.0 - 23.2.0): https://fmriprep.org/en/stable/ - Used for processing multiband, multiecho fMRI data.<br>SigmPlot v12.5: https://systatsoftware.com/sigmaplot/ - Used for fitting rise-to-maximum growth functions.<br>MIDB atlas v1.0: https://midbatlas.io/ - Provided as a data thresholding tool, written with custom HTML code.<br>ABCD Reproducible Matched Samples v1.0.0(ARMS) software - https://osf.io/7xn4f/ This tool was used to generating matched groups in ABCD.

For manuscripts utilizing custom algorithms or software that are central to the research but not yet described in published literature, software must be made available to editors/reviewers. We strongly encourage code deposition in a community repository (e.g. GitHub). See the Nature Research guidelines for submitting code & software for further information.

Data

Policy information about availability of data

All manuscripts must include a data availability statement. This statement should provide the following information, where applicable:

- Accession codes, unique identifiers, or web links for publicly available datasets
- A list of figures that have associated raw data
- A description of any restrictions on data availability

Neuroimaging data that were used to generate findings in this study are currently available from the NIMH Data Archive (NDA) upon approval with a data use agreement (https://nda.nih.gov/). All individual-specific maps for ABCD will be downloadable through the NIMH Data Archive (NDA) as well (pending approval). Investigators who wish to share individual-specific maps based on ABCD data, can do so via the ABCD-BIDS Community Collection (ABCCC; NDA Collection 3165)19,49. Probabilistic maps for each network are provided for each cohort separately are available at https://midbatlas.io/. The initial cohorts that are currently available at https://midbatlas.io/ are: HCP-D, ABCD,Dworetsky-Yale, Dworetsky-Darmouth,Dworetsky-MSC,and Dworetsky-HCP). The MIDB atlas which contains the probabilistic atlases is available from https://midbatlas.io

Field-specific reporting

Please select the one below that is the best fit for your research. If you are not sure, read the appropriate sections before making your selection.

☒ Life sciences ☐ Behavioural & social sciences ☐ Ecological, evolutionary & environmental sciences

For a reference copy of the document with all sections, see nature.com/documents/nr-reporting-summary-flat.pdf

Life sciences study design

All studies must disclose on these points even when the disclosure is negative.

Sample size	ABCD: n=11,572 participants enrolled. This sample size was collected to enable sufficient power to capture and analyze different patterns of substance use along with many other variables of interest (Volkow et al. 2018). n=2036 participants were excluded for excessive motion during scanning. Midnight scan club(MSC) : The MSC dataset collected only 10 subjects in part because the aim of the study was to acquire highly sampled data to examine within-subject reliability, within-subject task=related effects, and day-to-day subject variation. n=10 participants. HCP-D aimed to enroll 1300 children from ages 5 -21 (Sommerville et al. 2018). The total number of participants was selected to maximize the quantity of data acquired within the constraints of the project duration, available funding, and balancing between cross-sectional and longitudinal sessions. The final number of participants enrolled was n=652 participants, of which n=425 with low motion data. Only 5 participants have been collected with an extended duration fMRI collection in adolescents to be able to compare the the MSC dataset (MIDB subpop n=5).
Data exclusions	Unusable T1 data, Poor T1 registration, excessive motion in the MRI scanner.
Replication	Participants were randomly split into two groups (5786 each) such that that were matched along 10 variables: site location, age, sex, ethnicity, grade, highest level of parental education, handedness, combined family income, and exposure to anesthesia 48 (see Supplementary Table 1). Neural networks were replicated using independent methods (template matching, infomap, and non-negative matrix factorization). Probabilistic networks were also generated with additional data sets (See midbatlas.io). The probabilistic maps showed a nearly for each group were nearly identical (i.e. they were replicated). For the ABCD, and HCP datasets, each network map for a given method was only generated once. In analyses that examined an examination of the number of minutes required to produce reliable maps (i.e. the MIDB subpop

	and MSC), random time series sample was performed 10 times to generated to provide a range of similarity values. The sample at each time time interval showed a strong similarity to other random samplings of the same time interval.
Randomization	Participants were randomly split into two groups (5786 each), each group had similar demographic composition and retained a similar number of participants after removing participants without enough resting state data (group1 = 989, group 2 = 1047 participants excluded).
Blinding	Blinding is not relevant to this study. Specifically, the identity of the participants was anonymous to the researchers. Participants were randomly, but in a balanced manner based on balanced only demographics, placed into each ARM. Only after participants were placed into matched groups, were group-level BWAS performed.

Reporting for specific materials, systems and methods

We require information from authors about some types of materials, experimental systems and methods used in many studies. Here, indicate whether each material, system or method listed is relevant to your study. If you are not sure if a list item applies to your research, read the appropriate section before selecting a response.

Materials & experimental systems

n/a	Involved in the study
☒	☐ Antibodies
☒	☐ Eukaryotic cell lines
☒	☐ Palaeontology
☒	☐ Animals and other organisms
☐	☒ Human research participants
☒	☐ Clinical data

Methods

n/a	Involved in the study
☒	☐ ChIP-seq
☒	☐ Flow cytometry
☐	☒ MRI-based neuroimaging

Human research participants

Policy information about studies involving human research participants

Population characteristics	9-10 year old children recruited from across the United States with a racial and ethnic composition that reflects the U.S. population for the ABCD study. Full demographic table is provided in Supplementary table 1. HCP-D: 654 5-21 years old recruited at the University of Washington in St. Louis. MSC: 10 adults, average age 29 years. MIDB subpoplulation: 5 9-10 year olds.
Recruitment	ABCD Participants were largely recruited through the school systems with school selection informed by gender, race and ethnicity, socioeconomic status, and urbanicity. Procedures for school selection designed to mitigate selection biases, dynamic monitoring of the accumulating sample to correct deviations from recruitment targets, and a description of the recruitment procedures designed to foster a collaborative attitude between the researchers, the schools and the local communities were used. HCP-D participants were recruited from 4 different locations: Boston, Los Angeles, Minneapolis ,and St. Louis. For tracking and balancing recruitment goals biological sex report was used but data on self-perceived gender including non-binary options was acquired (Sommerville ety al. 2018). Sampling was designed to match the ethnic and racial diversity of the United States according to 2016 Census data (www.census.gov/quickfacts/fact/table/US/PST045216), diversity across socioeconomic status (SES) with a good faith effort to distribute SES over sex and race. In HCP-D, SES is computed using income-to-poverty ratio which is based on family income relative to poverty thresholds, adjusted for family size (Diemer et al., 2013). Three ranges were selected: approximately one third of the participants with income-to-poverty ratio in the 0–2.5 range, one third in the 2.5–5 range, and one third above 5. Some SES bias might arise from the fact that these data collection centers are located in major metropolitan areas, so they may lack representation from those that find physical, geographical, and financial and accessibility to participate.
Ethics oversight	Neuroimaging and behavioral data were collected in accordance with local IRBs at each institution. Analysis of pre-existing neuroimaging data was approved by the University of Minnesota Institutional Review board. In addition, ABCD JEDI Workgroups strives to ensure and promote justice, equity, diversity, and inclusion (JEDI) at all levels of ABCD, including within our measures and methods and our organizations and universities affiliated with ABCD, as well as to ensure that ABCD data are used and interpreted with the utmost integrity and in a way that prevents further stigmatization, marginalization, or injustice to individuals.

Note that full information on the approval of the study protocol must also be provided in the manuscript.

Magnetic resonance imaging

Experimental design

Design type	Analysis of connectivity used either resting-state fMRI or concatenated resting-state and task data.
Design specifications	Fro ABCD, During each scanning session, participants performed 4 types of scans: a resting state scan and 3 types of tasks, a Monetary incentive delay task, Spatial span task, and a nback task (Casey et al. 2018). https://abcdstudy.org/families/abcd-fmri-tasks-and-tools/ . Scanning for MSC, and HCP was performed only using resting state fMRI.
Behavioral performance measures	Analysis used previously-collected behavioral measures including the NIH toolbox tasks, assessments of mental health

Behavioral performance measures

using Kiddie Schedule for Affective Disorders and Schizophrenia (KSADS), and surveys of substance use, culture and environment.

Acquisition

Imaging type(s)

Structure, functional, diffusion (aquired but not used in this study).

Field strength

For ABCD, Sequences were harmonized across Siemens, Philips, and GE 3T scanners. For the MSC study a 3T scanner was used. The MIDB cohort was collected using a 3T MRI. The HCP-D cohort was also collected using a 3T MRI

Sequence & imaging parameters

Subjects underwent 25-45 minutes of pre-scan task compliance, localizer, 3d T1-weighted MRI (1mm isotropic, TR=(either 2500 or 6100 ms, TE=2-2.9 ms ,8o flip angle, 256 x 256 FOV), diffusion weighted images, 3d T2-weighted MRI (1mm isotropic, TR=2500 or 3200ms, TE=60–565ms, variable flip angle, 256 x 256 FOV), 1-2 runs of rs-fMRI (**2.4mm** isotropic, TR=800ms, TE=30, variable flip angle= 52o, 216 x 216 FOV), and a randomized order of monetary incentive delay (MID), stop signal task (SST), and emotional n-back (EN-back) tasks. For resting state scans: (TR =800 ms, TE =30 ms, flip angle = 90°, voxel size = 2.4 mm3, 60 slices).

For HCP-D fMRI scans were 21 minutes with the following parameters: fMRI scans are acquired with a 2D multiband (MB) gradient-recalled echo (GRE) echo-planar imaging (EPI) sequence (MB8, TR/TE = 800/37 ms, flip angle = 52°) and 2.0 mm isotropic voxels covering the whole brain (72 oblique-axial slices).

MSC was acquired with the following parameters: gradient-echo EPI sequence (TR = 2.2 s, TE = 27 ms, flip angle = 90, voxel size = 4 mm x 4 mm x 4 mm, 36 slices).

Area of acquisition

Brain only including brain stem.

Diffusion MRI ☐ Used ☒ Not used

Preprocessing

Preprocessing software

DCAN Processing pipeline was used for preprocessing and is a modified version of the Human Connectome project processing pipeline. Advanced Normalization Tools (ANTs) to perform denoising and N4 bias field correction, which significantly improves results for subjects scanned on General Electric (GE) and Philips scanners that tend to have more noise and are not always normalized following the scan.

Normalization

Advanced Normalization Tools (ANTs) to perform denoising and N4 bias field correction. Connectivity matrices were normalized within participants such that correlation values within/and between hemispheres, and the subcortex were transformed intro Z-scores prior to assigning networks.

Normalization template

MNI 305.

Noise and artifact removal

Advanced Normalization Tools (ANTs) to perform denoising and N4 bias field correction, which significantly improves results for subjects scanned on General Electric (GE) and Philips scanners that tend to have more noise and are not always normalized following the scan. Time courses were corrected using DCAN-BOLDproc (Feczko, E. et al. Adolescent Brain Cognitive Development (ABCD) Community MRI Collection and Utilities. bioRxiv 2021.07.09.451638 (2021) doi:10.1101/2021.07.09.451638). The method for signal regression has been previously described (Hermosillo et al., 2020). Briefly, resting state time courses (using surface registration for cortex and volume registration for subcortical gray matter) were detrended and further processed using mean whole brain, ventricle, and white matter signal as well as displacement on the 6 degrees of freedom, rigid body registration, their derivatives and their squares by regression (Ciric et al. 2017). In working with ABCD data, we have found that a respiratory artifact is produced within multi-band data (Fair et al., 2020). While this artifact occurs outside the brain, it can affect estimates of frame alignment, leading to inappropriate motion censoring. By filtering the frequencies (18.582 to 25.726 breaths per minute) of the respiratory signal from the motion realignment data, our respiratory motion filter produces better estimates of FD.

Volume censoring

Various thresholds were used to control for motion. Frames were removed if framewise displacement (FD) exceed threshold of 0.2 mm for example. If two censored frames were within 5 frames of each other then the frames in between are also removed. In addition, of the remaining frames, we removed frames that had excessively high standard deviations in the BOLD data (outliers using the Median absolute deviation). Within group reliability was tested using 10 minutes of randomly-sampled frames (fixed number of time points per subject) and also using all available motion-censored data (variable number of time points per subject).

Statistical modeling & inference

Model type and settings

For split half comparison with NMI, we used 2 sample tests. For between -group comparison of probabilistic maps, a bivariate Peasron's correlation was performed on each network. For brain wide association, we used correctional analyses. For within-network comparisons, we used 2 sample t-tests (two -tailed) independently for each network.

Effect(s) tested

In figure 4, using correlation between 1250 subjects from group1 and 3111 subjects from grou2, we calculated the effect size between the Gordon parcellation and the MIDB parbabilistic atlas and we found effect sizes of PC1=0.909, PC2=1.605,PC3=1.865

Specify type of analysis: ☒ Whole brain ☐ ROI-based ☐ Both

Statistic type for inference
(See Eklund et al. 2016)

For group level analyses, Inferences based significance are not drawn. Instead corresponding maps are correlated (using Pearson's correlation). For within-subject data Normalized mutual information is measured against a null distrubution (non-self normalized mutual information)

Correction

Correlations for probabilistic maps were conducted between group1 and group2 for each network separately. We did not apply a correction to these data. Statistical comparisons of the within network connectivity between the Gordon parcellation and the MIDB probabilistic parcellation were tested independently, and no correction was applied.

Models & analysis

n/a	Involved in the study
☐	☒ Functional and/or effective connectivity
☐	☒ Graph analysis
☒	☐ Multivariate modeling or predictive analysis

Functional and/or effective connectivity

Functional connectivity was performed with Fisher Z matrices. These matrices were then Z-scored as described above (within and between brain structures). For within-subject data Normalized mutual information is measured against a null distribution (non-self normalized mutual information).

Graph analysis

Infomap uses a weighted graph from Fisher Z matrices. Infomap assigns networks based on minimum information necessary describe flow in the network. No particular graph metrics are directly compared, only group assignments are used.
